# Machine learning improves SNP microarray performance in challenged samples

**DOI:** 10.1093/bioadv/vbag086

**Published:** 2026-03-21

**Authors:** Austin Chiao, Benjamin Crysup, Jonathan L King, Michael D Coble, August E Woerner

**Affiliations:** Center for Human Identification, University of North Texas Health Fort Worth, Fort Worth, TX 76107, United States; Department of Microbiology, Immunology and Genetics, University of North Texas Health Fort Worth, Fort Worth, TX 76107, United States; Center for Human Identification, University of North Texas Health Fort Worth, Fort Worth, TX 76107, United States; Department of Microbiology, Immunology and Genetics, University of North Texas Health Fort Worth, Fort Worth, TX 76107, United States; Center for Human Identification, University of North Texas Health Fort Worth, Fort Worth, TX 76107, United States; Center for Human Identification, University of North Texas Health Fort Worth, Fort Worth, TX 76107, United States; Department of Microbiology, Immunology and Genetics, University of North Texas Health Fort Worth, Fort Worth, TX 76107, United States; Center for Human Identification, University of North Texas Health Fort Worth, Fort Worth, TX 76107, United States; Department of Microbiology, Immunology and Genetics, University of North Texas Health Fort Worth, Fort Worth, TX 76107, United States

## Abstract

*Summary:* SNP microarrays provide a cost-effective genotyping method used in various scientific disciplines. Sample costs vary from tens to hundreds of dollars, storage costs are comparatively reasonable, and analysis methods easily scale to large sample sizes. However, microarrays are designed to be used with high quality samples rather than low-quantity DNA inputs. To deal with this, when working with challenged samples uncertainty must be properly accounted for. Rather than calling crisp genotypes when data are uncertain, it is better to represent them probabilistically. This approach can cleanly feed into tools that directly consider likelihoods while remaining compatible with tools expecting hard calls by removing uncertain genotype calls. Several machine learning algorithms were used to estimate genotypes and genotype likelihoods generated from Illumina Omni5-4 microarray data, and the results were compared. While neural networks and XGBoost were both performant, XGBoost appears to generalize better across sample types generated on the Omni5-4 chips (generalization between technologies awaits further examination). Further, it can more directly produce an estimate of genotype quality (as opposed to scores), a feature that has been lacking in microarray analysis.

## 1 Introduction

SNP microarrays are a deceptively simple platform. In brief, fluorescently labeled olignonucleotide probes target (typically two) potential alleles (A and B, A being the relevant reference at the site and B the alternative allele) at a given site. The intensity values corresponding to each allele are then used to estimate the most likely genotype (AA, AB or BB) using something like the GenTrain algorithm found in GenomeStudio (as well as Illumina Dragen). GenTrain performs well with high quality samples. However, as sample quality wanes, so does performance, often in a punctuated fashion (Russell *et al.* 2023). Challenged samples often have low call rates and reduced accuracy, which has obvious impacts on downstream analyses [e.g. kinship classification (Nielsen *et al.* 2011, 2012, de Vries *et al.* 2022, Turner *et al.* 2022)]. While low quality samples ought to yield low quality results, it is possible current algorithms make the situation worse by leaving information on the table: how much of the drop off is due to inefficient genotyping?

GenTrain relies on unsupervised techniques to cluster A/B intensities into three distinct cluster groups while noting certain quality control measures. Unsupervised algorithms are an atypical solution to the problem; genotype estimation is naturally a question of prediction, either as a supervised machine learning problem (Poplin *et al.* 2018) or using maximum likelihood (Li *et al.* 2009). A natural shortcoming of GenTrain is that it only considers local information (each site is genotyped assuming independence, which is unlikely to hold in lower quality samples) and it provides a single categorical prediction (the most likely genotype) along with an uncalibrated “quality” score (GenCall). However, GenTrain also provides several relevant summary statistics, suggesting that this widely adopted algorithm may be adapted (*post hoc*) to better suit challenged samples using supervised machine learning.

In low quality samples, knowing the exact genotype may not be strictly important so long as uncertainty is appropriately expressed. Compromised samples naturally lend themselves to probabilistic frameworks, which rely on the genotype likelihood rather than called genotypes (Nielsen *et al.* 2011, Skotte *et al.* 2012, Korneliussen *et al.* 2014, Nguyen *et al.* 2023). Many uncertain genotypes can be incorporated through principal component analysis (PCA) (Fumagalli *et al.* 2013, Fumagalli *et al.* 2014, Meisner and Albrechtsen 2018), for example. Alternatively, genotype imputation methods can use genotype likelihoods and accompanying linkage disequilibrium (LD) maps to estimate genotypes with surprising efficiency and performance (Pasaniuc *et al.* 2012, Cai et al. 2015, Gilly *et al.* 2016). The same probabilistic frameworks may also be applicable to SNP microarrays if the data can be represented in an appropriate way. Machine learning techniques can be used to learn complex relationships in data. Some techniques, like Neural Networks (NN), serve as universal approximators (Hornik *et al.* 1989) and have been successfully applied to empirically learn genotype likelihoods in whole genome sequencing (WGS) data (Poplin *et al.* 2018).

**Figure 1:**
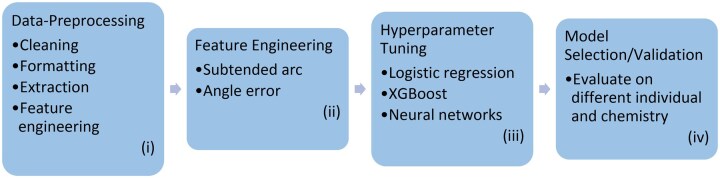
Overview of project flow. The workflow for the study is as follows: (i) Microarray data preprocessing. (ii) Feature engineering. (iii) Hyperparameter tuning. (iv) Model selection/validation.

Machine learning techniques may improve the performance of SNP microarrays, perhaps allowing (relatively) poor quality samples to be successfully genotyped. We explored several classes of supervised machine learning models to determine which is the most performant across a dilution series. The chosen model was then retrained and tested for its performance. Several filtering steps were introduced to improve accuracy and call rate. The proposed method in this study also generates probabilities which can be used as a quality filter. The results were compared against the genotypes obtained directly from GenomeStudio. Figure 1 shows the overall steps involved in the study.

## 2 Methods

### 2.1 DNA samples

DNA samples were collected from eight individuals (Fig. 1 and Table 1, available as supplementary data at *Bioinformatics Advances* online) and genotyped on Illumina Omni 5-4 SNP microarray chips according to manufacturer protocol. Samples were evaluated in a titration series, considering DNA inputs of 50 ng, 1 ng, 0.5 ng, 0.1 ng, 0.05 ng, and 0.01 ng. Cell line DNA samples were obtained from the NIGMS Human Genetic Cell Repository at the Coriell Institute for Medical Research.

**Table 1:** Raw summary statistics exported from Genome Studio.

Feature	Purpose
GenTrain score	Quality metric for the cluster
GenCall score	Quality metric for the call
R	signal intensity values
θ	measurement of the B signal intensity (relative to A)
Cluster separation	Separation between clusters for the SNP

### 2.2 Microarray chemistry

SNP microarrays were used to target large numbers of biallelic SNPs. Prepared cluster files were then used to map the red/green intensities (Steemers *et al.* 2006) to AA, AB, and BB genotypes using the GenCall algorithm. Raw summary statistics were collected (Table 1). SNP calls with a GenCall score below the default threshold of 0.15 were removed.

### 2.3 Machine learning

#### 2.3.1 Data preprocessing and feature engineering

Data filtering, visualizations, and statistics were performed in the R programming language (R Foundation, https://www.r-project.org/), using the ggplot2 package (Wickham 2016). Feature engineering and machine learning were conducted in Python (Python Software Foundation, https://python.org/) with aid of the following libraries: Pandas (McKinney 2010), Scikit-learn (Pedregosa *et al.* 2011), Keras (Chollet 2015), Numpy (Harris *et al.* 2020), Scipy (Virtanen *et al.* 2020), and Matplot (Hunter 2007).

Several quality control steps were taken. Markers from non-autosomal chromosomes were removed (X, Y, and mitochondrial), as well as those with indeterminate chromosome data (labeled Chr0). SNPs with invalid identifiers (dashes or missing) were also removed. Only unique SNP names were retained in the dataset, and any duplicates were removed.

Key summary statistics (Table 1) were combined, normalized, and augmented to improve model performance. Informally, the angle errors describe the extent to which θ deviates from the 0°, 45°, and 90° (i.e. the respective expected value of AA, AB, BB genotypes assuming equal probe efficiency), while the subtended arc describes the length associated with those angle errors. The signal variance and mean signal intensity were also extracted and added to the features. All features were normalized (z-scores).

The angle error (A) is defined as:


(1) AAA=θ

(2) ABB=(π2-θ)

(3) AAB=(π4-θ)

while the subtended arc is defined as:


(4) SAA=Rθ 

(5) SAB=R(π4-θ)

(6) SBB=R(π2-θ)

The 50 ng samples were designated as ground truth, and no calls (across DNA inputs) were removed. Genotype accuracy was evaluated by matching ground truth genotypes with the corresponding SNPs at the lower DNA inputs.

#### 2.3.2 Machine learning algorithms

Three algorithms were investigated: regularized multinomial logistic regression (RMLR), XGBoost, and neural networks (NN) (James *et al.* 2013, Chen *et al.* 2015).

##### 2.3.2.1 Regularized multinomial logistic regression

Several RMLR hyperparameters were tuned, including the regularization strength (C), the penalty type, and the L1 ratio for elastic net regularization.

##### 2.3.2.2 XGBoost

XGBoost, a tree-based method, was also employed (Chen *et al.* 2015). XGBoost was set to perform multiclass categorical classification while producing classification probabilities, using the cross-entropy of the softmax of the scores as the objective function. The following hyperparameters were tuned: number of estimators, learning rate, threshold controlling tree complexity, sample size split threshold, and how much the training data was sub-sampled.

##### 2.3.2.3 Neural network

The third model explored in the study was a NN composed of three hidden layers. The activation function of the hidden nodes was Leaky ReLU and that of the output layer was softmax. Training was done via Adam with sparse categorial entropy as the loss function.

#### 2.3.3 Machine learning optimization

Test and training sets were created by randomly partitioning the data by individual. Seven individuals were used for cross validation and hyperparameter tuning, while model performance was assessed on the remaining individual (NA07046) (test data set). Hyperparameter tuning was performed using seven-fold cross validation (by individual). Hyperparameters were optimized using a grid search, using the negative log loss as the scoring function.

#### 2.3.4 Machine learning strategy

Machine learning was conducted in two phases. In the first phase (model selection), the best category of machine learning models (RMLR, XGBoost, or NN) was selected. To address class imbalance, the training dataset was down sampled to create an equal number of correct and incorrect genotypes (noting that 93.5% of sites were correctly genotyped in the training dataset). Down sampling was chosen to reduce the computational burden of hyperparameter optimization. Informally, this approach sets a flat prior probability on correctness and provides a natural way to interpret key summaries (relative to the null random classifier) while being computationally efficient.

The best models of each category were compared (between machine learning model types) based on several performance metrics, including recall, precision, and accuracy. A category was selected and a final model was then trained on the full training dataset with hyperparameter optimization. The final model was trained without subsampling: class imbalance was addressed by reweighting classes. Informally, the final model considers all training data consisting of seven individuals (computationally intensive, but only occurs once), whilst ensuring a flat prior probability on genotypes (emulating a likelihood function). The model was also separately trained on a modified training set where unrelated individuals (7013 and 7035) were excluded to test if familial relationships could cause data leakage (Table 2, available as supplementary data at *Bioinformatics Advances* online). The model appeared to perform similarly to the model trained on seven individuals (see Tables 7–10 and 16–23, available as supplementary data at *Bioinformatics Advances* online).

**Table 2:** The genotyping performance of genome studio.

DNA input	Accuracy	Relative call rate
50 ng	—	99.48%
1 ng	99.93%	98.41%
0.5 ng	96.45%	97.33%
0.1 ng	95.65%	94.85%
0.05 ng	93.94%	95.39%
0.01 ng	71.28%	78.46%

Machine learning was performed using Scikit-learn (v1.4.2) in Python (v3.10.7).

#### 2.3.5 Filtering

A Phred-scaled genotype quality filter was applied to the predicted SNPs from the models, with four minimum thresholds under consideration: 0, 10, 20, and 30. Phred genotype quality (Q) scores were calculated as $Q=-10Log_{10}(P)$, where *P* is the probability of an error in a base call taken from the classifier (Ewing and Green 1998). The probabilities from XGBoost were not calibrated: as this work uses them as simple thresholds this is acceptable, though for other applications calibration should be carefully considered. Compared to GenomeStudio, Q scores were compared to GenCalls. As GenCalls are uncalibrated, matching sets (Q vs. GC scores, set to the have equal cardinality) were determined by rank. Q scores were ranked in increasing scores with the same rankings applied to the GC scores.

An additional filter based on concordance was also considered. Namely, a genotype call was considered concordant if the predicted call matched between GenomeStudio and the *post hoc* classifier (e.g. XGBoost). The final model’s AB F1 scores and accuracy were significantly higher than those provided by GenomeStudio (*P* = 1.907e-06, 95% CI: −0.13961329 to 0.08824321, *P* = 1.907e-06, 95% CI: −0.0314 to −0.0213, paired sign test).

#### 2.3.6 Kinship estimation

Kinship was estimated to determine if the inferred genotypes also improve downstream biological inferences. Plink 2.0 (Purcell *et al.* 2007) was used to estimate the King-Robust (Manichaikul *et al.* 2010) kinship coefficient. Actual kinship coefficients were taken from 50 ng-50 ng comparisons using genotypes provided by GenomeStudio, while expected coefficients were derived from the pedigree.

## 3 Results

### 3.1 Preprocessing

Twelve microarray runs were performed, characterizing 4.3 million SNPs (per sample per run), providing 208 million records in total (eight individuals × six dilutions). After applying quality controls, 155 million autosomal SNPs were retained for analysis.

### 3.2 Genotyping results

The mean genotype accuracy and call rate from GenomeStudio across DNA inputs were tabulated (Table 2). Call rates for each individual was also calculated (Table 15, available as supplementary data at *Bioinformatics Advances* online). Call rates were generally high, excepting the lowest DNA input (0.01 ng). Genotype accuracy, however, dropped gradually throughout the series (Table 2). The absolute call rate, relative call rate, and accuracy are calculated with the following equations.


(7) Absolute call rate=Total sites-Uncalled sitesTotal sites on Omni 5-4 (excluding non-autosomal chromosomes)

(8) Relative call rate=Total sites for each DNA input-Uncalled sitesTotal sites for each DNA input

(9) Accuracy=Total sites-(Uncalled sites+Wrong calls)Total sites-Uncalled sites

### 3.3 Signal variance and intensity

**Figure 2:**
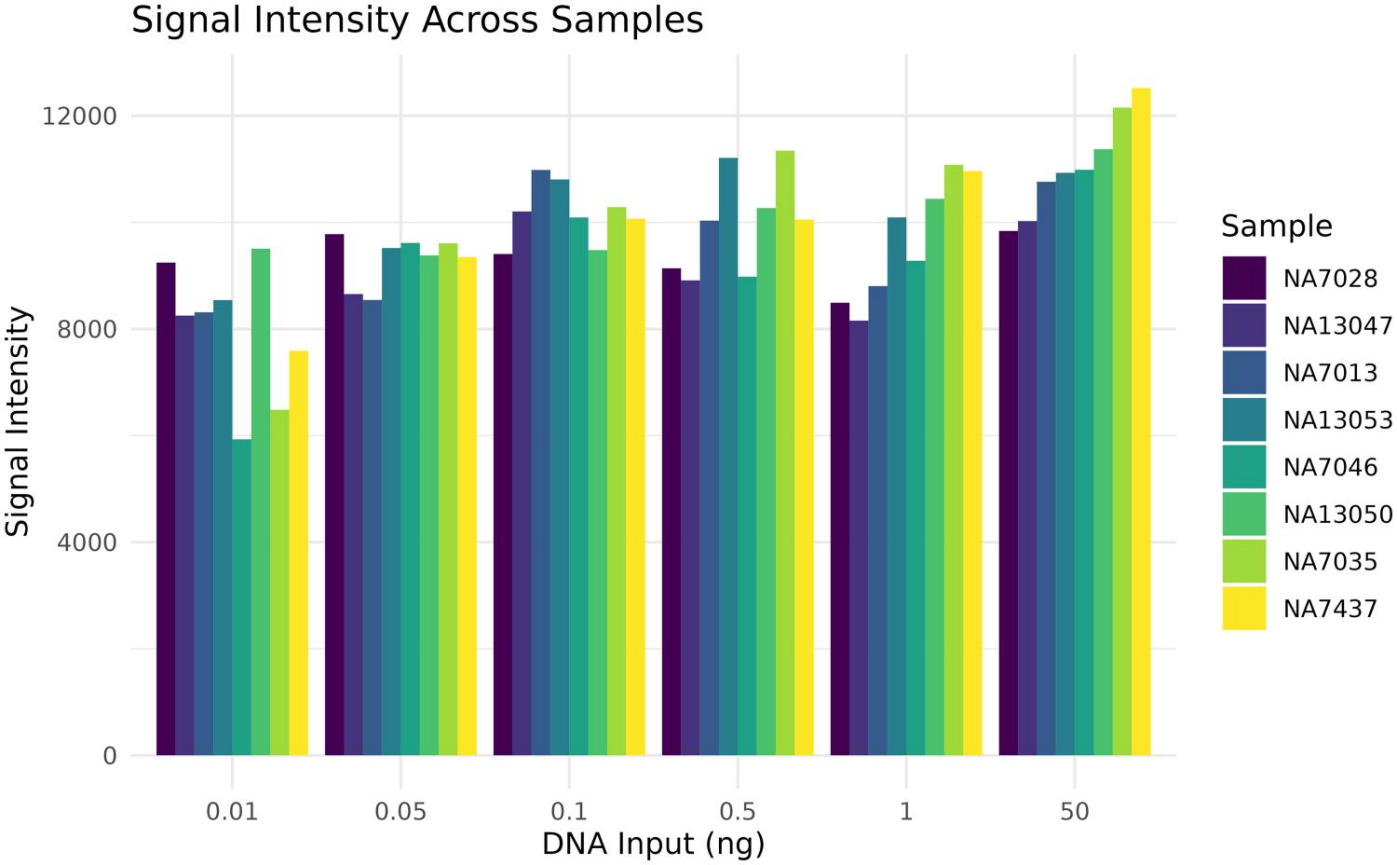
Mean signal intensities across DNA inputs. Different DNA inputs (x-axis) have corresponding signal intensities (y-axis) as measured in different individuals (colors).

**Figure 3:**
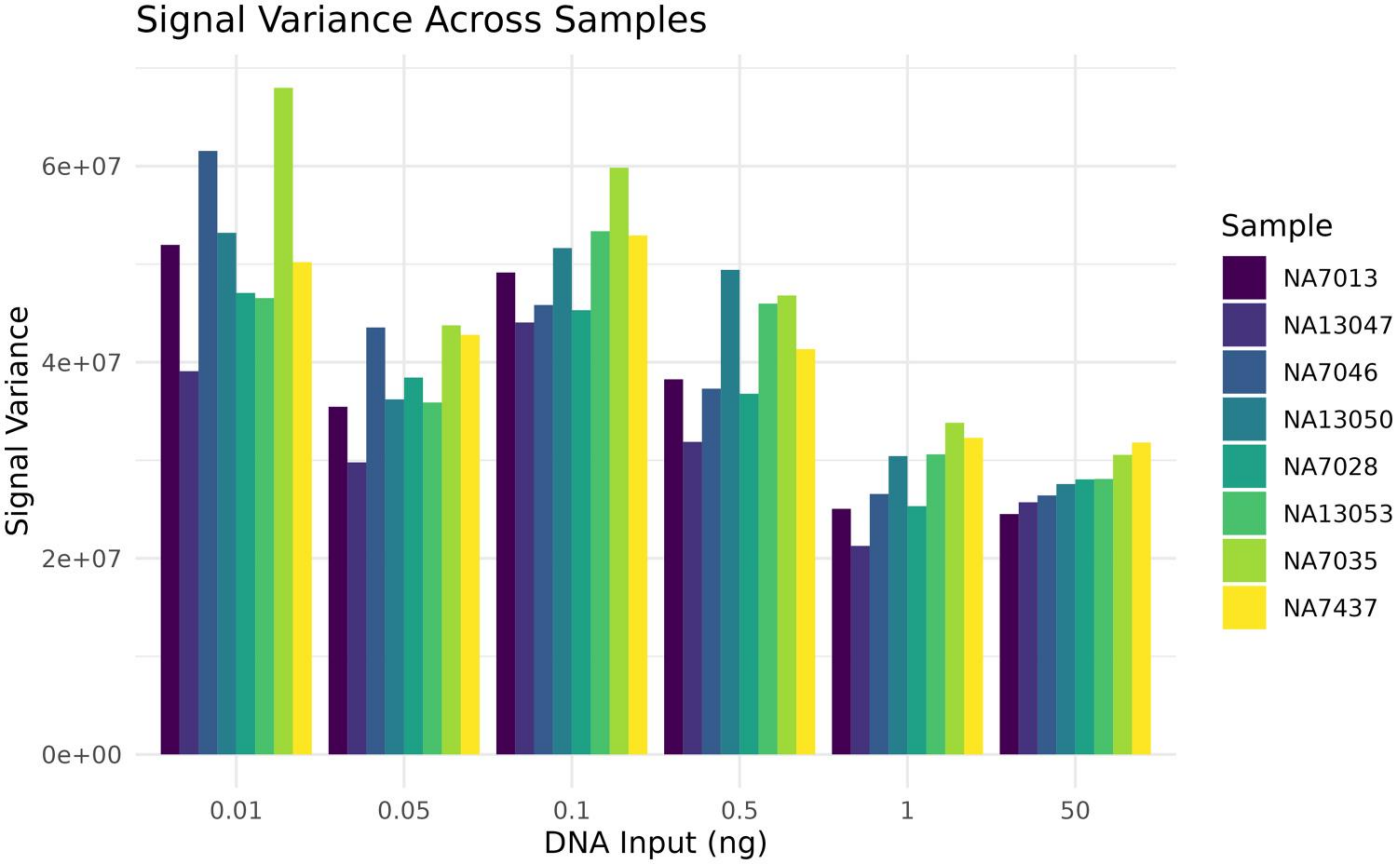
Mean signal variance across DNA inputs. The signal variance (y-axis) corresponds to the DNA inputs (x-axis) measured in different individuals (colors).

The raw signal intensity means (Fig. 2), and variances (Fig. 3) were calculated. The mean signal intensity appears to be positively correlated with DNA input (Pearson *r* = 0.48) suggesting some level of quantitation to the assay. The relationship between DNA input and signal variance appears similar (Pearson *r* = −0.49). First principles suggest that both the mean (positive correlation, less DNA gives less signal intensity) and the variance (negative correlation, less DNA gives more variability in intensity) should be predictive of DNA input (Huber *et al.* 2002). As such, both features were considered in our ML models as proxies for sample quality and/or quantity.

### 3.4 Training

The three models were trained on seven individuals with group *k*-fold cross validation. Each of the models underwent hyperparameter tuning. A grid search showed that an elastic net with the L1 ratio set to 0.001 for RMLR was optimal. Thousand estimators with a max depth of 100 and subsampling of 0.8 was determined to be the most optimal for XGBoost. The learning rate for NN was set at 0.01 with 50 and 100 neurons for the first and second hidden layers respectively.

### 3.5 Feature importance

RMLR is both machine learning technique and statistical inference tool (James *et al.* 2013). While the interpretation of RMLR coefficients can be complicated, the normalized coefficients can indicate feature importance (Table 3). The magnitude of many of the coefficients appears to be large. For example, while GenomeStudio treats the GenCall score as the primary indicator of genotype quality (e.g. in Illumina’s GTC2VCF software, the GenCall score is literally converted into a Phred-scaled genotype quality score), other features such as R, subtended arcs, and angle errors showed larger coefficients across genotype classes, indicating other features may be more predictive of genotype quality.

**Table 3:** Regression coefficients from RLMR.

Features	A A	A B	B B
GenCall	−0.29	0.62	−0.33
GenTrain	1.14	−0.79	−0.35
Cluster separation	0.15	−0.72	0.57
R	−3.2	1.94	1.26
X	−3.38	0.64	2.75
Y	1.07	−1.79	0.73
Theta	−1.01	0.09	0.91
Allele AA	−1.67	0.92	0.75
Allele BB	1.79	−0.2	−1.59
Allele AB	0.46	−1.1	0.64
Raw signal intensity	0.86	0.44	−1.31
45 Theta	1.91	−0.7	−1.21
90 Theta	1.46	−0.4	−1.06
Absolute signal intensity	0.02	−0.49	0.47
$S_{AA}$	−7.74	2.47	5.27
$S_{AB}$	2.71	0.57	−3.29
$S_{BB}$	5.23	−0.95	−4.28
Signal intensity mean	0.56	−0.43	−0.13
Signal variance	−0.81	0.48	0.33
Angle error	−3.94	2.39	1.55
Intercepts	0.58	−0.39	−0.19

### 3.6 Model performance

While RMLR is a relatively fast method, its performance is markedly worse than both XGBoost and NN (Tables 3–6 and Fig. 2, available as supplementary data at *Bioinformatics Advances* online). Surprisingly, RMLR performs especially poorly when the DNA input is large if no quality threshold is considered.

**Table 4:** Absolute call rates (4 198 873 SNPs were genotyped).

Input	Phred filter	RMLR	XGBoost	NN
1 ng	10	92.80%	98.08%	96.52%
	20	82.48%	97.92%	90.62%
	30	67.61%	97.64%	73.77%
0.05 ng	10	81.92%	92.40%	88.84%
	20	58.74%	87.12%	70.14%
	30	45.10%	81.20%	53.79%

**Table 5:** Relative call rates.^a^

Input	Phred filter	RMLR	XGBoost	NN
1 NG	10	94.57%	99.95%	98.36%
	20	84.04%	99.78%	92.34%
	30	68.89%	99.49%	75.17%
0.05 ng	10	86.56%	97.63%	93.87%
	20	62.07%	92.05%	74.12%
	30	47.65%	85.79%	56.84%

a For 1 ng 4 120 603 SNPs were genotyped. At 0.05 NG 3 973 861 SNPs were genotyped.

**Table 6:** Model F1 score of AB without concordance.^a^

	Phred filter	RMLR	XGBoost	NN
1 ng	0	76.06% (−23.71%)	99.72% (−0.05%)	94.43% (−5.34%)
	10	83.68% (−16.15%)	99.80% (−0.03%)	96.45% (−3.39%)
	20	93.56% (−6.30%)	99.91% (+0.05%)	98.61% (−1.25%)
	30	97.00% (−2.91%)	99.95% (+0.04%)	99.12% (−0.78%)
0.05 ng	0	63.59% (−10.88%)	80.12% (+5.65%)	74.34% (−0.13%)
	10	76.13% (−0.28%)	83.35% (+6.94%)	90.91% (+14.50%)
	20	91.47% (+10.98%)	89.11% (+8.62%)	89.45% (+8.96%)
	30	96.56% (+13.74%)	92.15% (+9.33%)	94.89% (+12.07%)

a The percentage point difference in parenthesis measures the difference between classifier and GenomeStudio. Positive values indicate better performance.

XGBoost and NNs are two nonlinear methods that maintain higher accuracy throughout the titration series (relative to RMLR). For example, XGBoost is ∼99.9% accurate at 1 ng, but at the lowest titration level, GenomeStudio was slightly more accurate (71.3% vs. 68.3%, before filtering), though neither technique is performant with so little DNA. NN performed similarly to XGBoost, however accuracy wanes when more DNA is considered (97.5% at 1 ng, vs. 99.9% for XGBoost and GenomeStudio), especially in the absence of a genotype quality filter.

**Figure 4:**
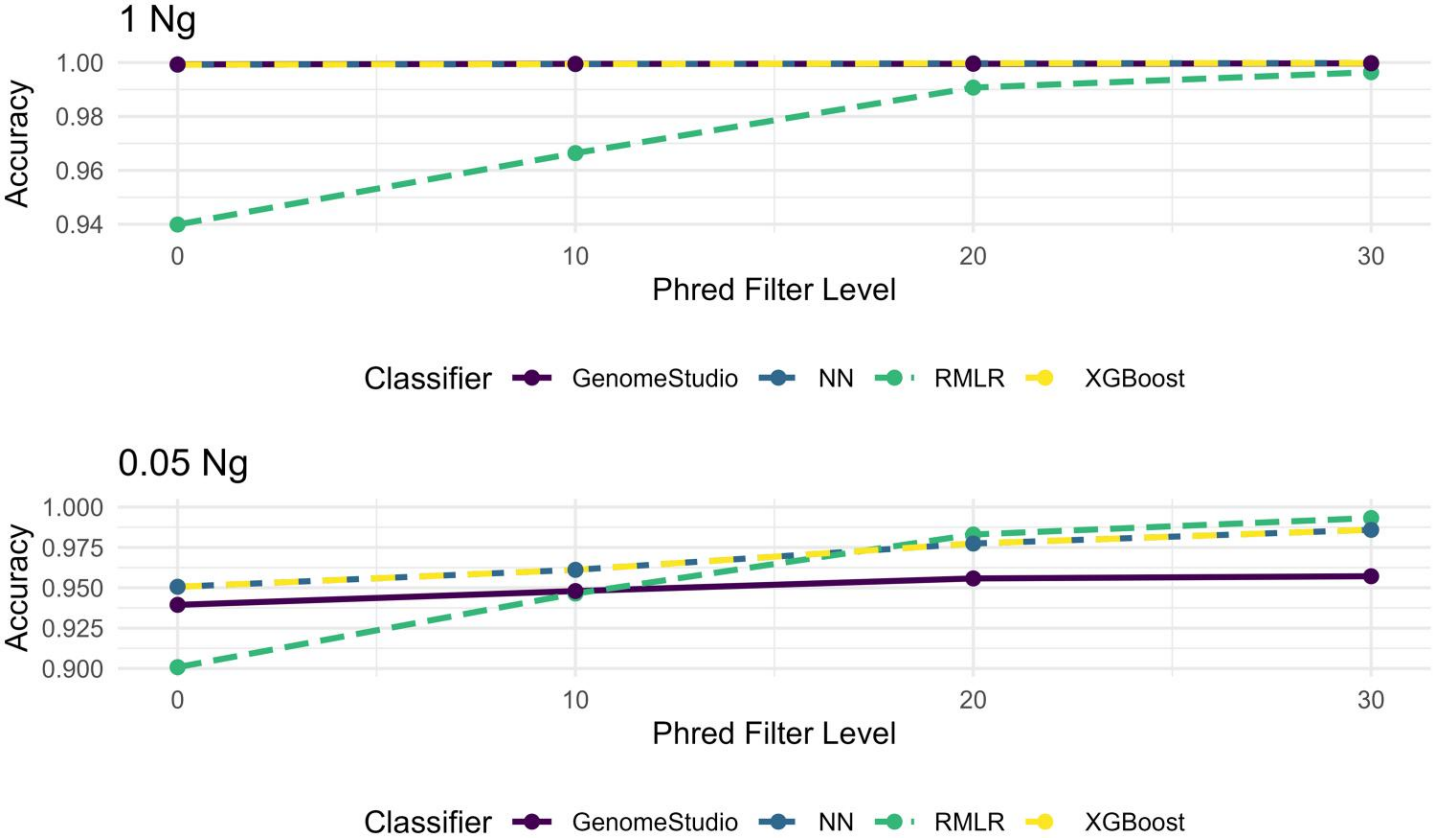
Accuracy comparing the different classifiers at different thresholds. The top figure is at 1 ng and the bottom figure is at 0.05 ng.

Genotype quality filters with Phred thresholds of 10, 20, and 30 were applied. Matching filters were applied to the results from GenomeStudio, noting that samples were matched on rank as the GC score does not readily convey a probability. Not surprisingly, more stringent criteria provide more accurate genotypes, excepting cases where the genotyping was already performant (e.g. XGBoost, 1 ng) (Fig. 4). However, more stringent thresholds also reduce call rates (Tables 4 and  5), especially at lower DNA inputs. For RMLR and NN roughly half of the SNPs were filtered out, leaving behind 1 893 598 and 2 258 556, respectively. XGBoost retained 3 409 343 SNPs.

**Table 7:** Model accuracy without concordance filter applied.^a^

	Phred filter	RMLR	XGBoost	NN
1 ng	0	93.99% (−5.94%)	99.92% (−0.01%)	98.44% (−1.49%)
	10	96.64% (−3.31%)	99.94% (−0.01%)	99.06% (−0.89%)
	20	99.07% (−0.88%)	99.97% (+0.02%)	99.68% (−0.27%)
	30	99.64% (−0.33%)	99.98% (+0.01%)	99.82% (−0.15%)
0.05 ng	0	90.08% (−3.86%)	95.06% (+1.12%)	93.94% (−0.00%)
	10	94.64% (−0.15%)	96.11% (+1.32%)	96.12% (+1.33%)
	20	98.30% (+2.72%)	97.74% (+2.16%)	98.20% (+2.62%)
	30	99.32% (+3.61%)	98.59% (+2.88%)	99.25% (+3.54%)

a The percentage point difference in parenthesis measures the difference between classifier and GenomeStudio. Positive values indicate better performance.

Accuracy alone presents an incomplete picture of model performance. Amongst other things, it does not characterize performance across genotype classes. Another metric, the F1 score, provides additional information. The AB class is likely to be the hardest to classify as it is a mixture of two probes and a combination of two signals. Consequently, heterozygosity of challenged samples may be taken as a measure of signal correctness (Russell *et al.* 2023). At the highest DNA input, RMLR’s AB F1 score is 76%, compared to 99% for GenomeStudio, while XGBoost and NN have F1 scores of 99% and 94%, respectively (Table 6). At the lowest DNA input, RMLR’s AB F1 scores are 64%, compared to 74% for GenomeStudio, while XGBoost and NN have F1 scores of 80% and 74%, respectively (Table 6). RMLR does not perform as well as GenomeStudio when trying to make predictions on the AB class.

### 3.7 Model choice

XGBoost was found to be the most performant model. XGBoost generalized better across DNA inputs and across genotype class (Tables 3–6, available as supplementary data at *Bioinformatics Advances* online) relative to either NN or RMLR. Compared to the other two methods, XGBoost had higher accuracy (95.06%) and F1 scores (80.12%) in the heterozygous classes along with higher call rates (Tables 4–7, Tables 11 and 12, available as supplementary data at *Bioinformatics Advances* online).

**Figure 5:**
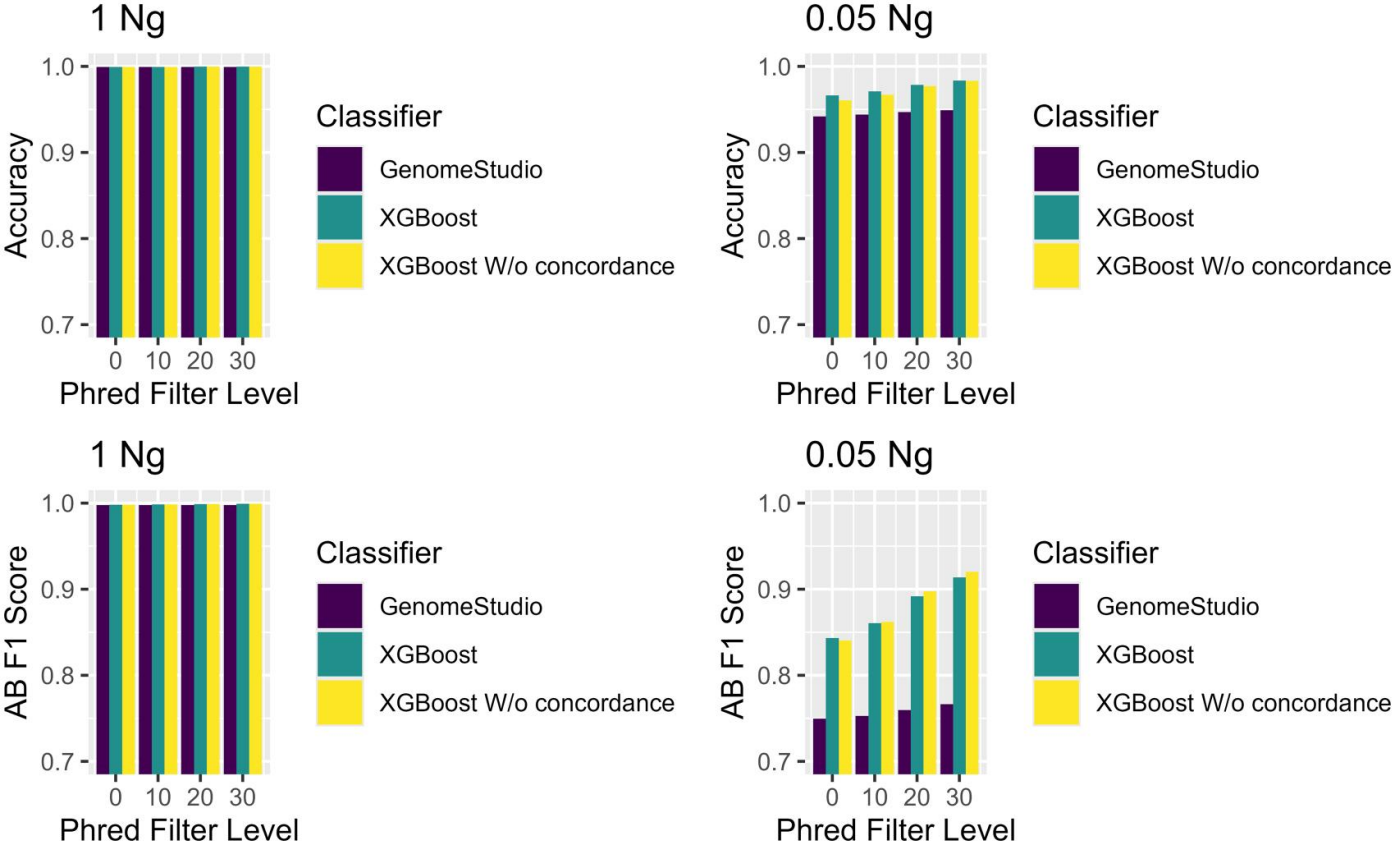
Accuracy and AB F1 score between GenomeStudio, XGBoost with a concordance filter, and XGBoost without a concordance compared at different DNA input and Phred quality filter threshold.

XGBoost was then trained with the full training set with hyperparameter tuning. Compared to GenomeStudio at 1 ng, both models achieved a 99.9% accuracy (Table 8, Fig. 4, available as supplementary data at *Bioinformatics Advances* online). However, starting at 0.5 ng, XGBoost began to outperform GenomeStudio (Table 7  and Fig. 4, available as supplementary data at *Bioinformatics Advances* online). The F1 scores for the heterozygous class and homozygous class were higher in XGBoost than in GenomeStudio (Tables 8–10, available as supplementary data at *Bioinformatics Advances* online) noticeably starting from 0.5 ng. The genotype quality filters showed a similar trend. As the filtering threshold became more stringent, the accuracy of XGBoost increased (Fig. 5, Fig. 3, available as supplementary data at *Bioinformatics Advances* online). For example, at 0.05 ng the accuracy of XGBoost with and without the concordance filter remained about the same (Table 8). At 0.01 ng with no threshold, XGBoost achieved an accuracy of 86.9% compared to 76.3% with GenomeStudio (Table 7, available as supplementary data at *Bioinformatics Advances* online). At a threshold of 30, the accuracy at 0.01 ng increased to 99.7% (Table 7, available as supplementary data at *Bioinformatics Advances* online) but the call rate dropped to about half (Table 11, available as supplementary data at *Bioinformatics Advances* online). The same general trend can be seen in the F1 Score for the AB class (Fig. 5; Fig. 3, available as supplementary data at *Bioinformatics Advances* online).

**Table 8:** XGBoost accuracy without and with concordance applied at 0.05 ng.^a^

	Phred filter	Without concordance	With concordance
1 ng	0	99.94% (+0.01%)	99.95% (+0.02%)
	10	99.95% (+0.02%)	99.95% (+0.02%)
	20	99.96% (+0.03%)	99.96% (+0.03%)
	30	99.97% (+0.04%)	99.97% (+0.04%)
0.05 ng	0	96.05% (+1.85%)	96.65% (+2.44%)
	10	96.71% (+2.32%)	97.09% (+2.70%)
	20	97.71% (+3.04%)	97.85% (+3.17%)
	30	98.31% (+3.40%)	98.36% (+3.44%)

a The percentage point difference in parenthesis measures the difference between classifier and GenomeStudio. Positive values indicate better performance.

**Table 9:** AB F1 score without and with concordance applied at 0.01 ng.^a^

	Phred filter	Without concordance	With concordance
1 ng	0	99.82% (+0.05%)	99.82% (+0.05%)
	10	99.84% (+0.07%)	99.84% (+0.07%)
	20	99.88% (+0.11%)	99.88% (+0.11%)
	30	99.91% (+0.14%)	99.91% (+0.14%)
0.05 ng	0	84.05% (+9.10%)	84.34% (+9.39%)
	10	86.20% (+10.93%)	86.06% (+10.79%)
	20	89.76% (+13.81%)	89.16% (+13.21%)
	30	92.01% (+15.38%)	91.37% (+14.74%)

a The percentage point difference in parenthesis measures the difference between classifier and GenomeStudio. Positive values indicate better performance.

**Table 10:** XGBoost absolute call rates.^a^

Input	Phred filter	XGBoost W/O	XGBoost
1 ng	10	98.12%	98.11%
	20	98.10%	98.09%
	30	98.07%	98.07%
0.05 ng	10	93.28%	90.64%
	20	90.53%	88.90%
	30	87.94%	86.84%

a A total of 4 198 873 SNPs were genotyped excluding non-autosomal chromosomes.

**Table 11:** XGBoost relative call rates.^a^

Input	Phred filter	XGBoost W/O	XGBoost
1 ng	10	99.99%	99.99%
	20	99.96%	99.97%
	30	99.93%	99.95%
0.05 ng	10	98.56%	99.22%
	20	95.66%	97.32%
	30	92.92%	95.05%

a A total of 4 120 603 SNPs were classified at 1 ng and 3 973 861 SNPs at 0.05 ng.

After applying a genotype quality filter, a concordance filter was also introduced. The concordance filter removes genotype predictions from the machine learning model that differs from the original calls from GenomeStudio. With the filter applied, XGBoost demonstrated slight improvements in accuracy at all titration levels, with the starkest improvements at the lowest DNA input. For example, at 0.05 ng the accuracy of XGBoost increased from 96% to 98% when increasing the Phred filter from 0 to 30 (Table 8). At 0.01 ng with a threshold of 0, XGBoost with concordance achieved an accuracy of 86.9% compared to 79.52% (Table 7, available as supplementary data at *Bioinformatics Advances* online) without concordance, though this difference disappeared at the most stringent filter. Interestingly the AB F1 score was slightly lower when a concordance filter was applied for XGBoost at 0.05 ng when compared to the model without the concordance filter (Table 9). At 0.01 ng without a genotype quality filter, XGBoost with a concordance filter only had a 38.92% F1 AB score while the model without the concordance filter achieved a 50.23% F1 AB score (Table 8, available as supplementary data at *Bioinformatics Advances* online). This gap was later reduced with more stringent levels of genotype quality filter. Call rates were also observed to decrease as the genotype filter became more stringent while call rates with and without the concordance filter were about the same (Table 10 and  11; Tables 13 and 14, available as supplementary data at *Bioinformatics Advances* online).

### 3.8 Kinship coefficient

**Figure 6:**
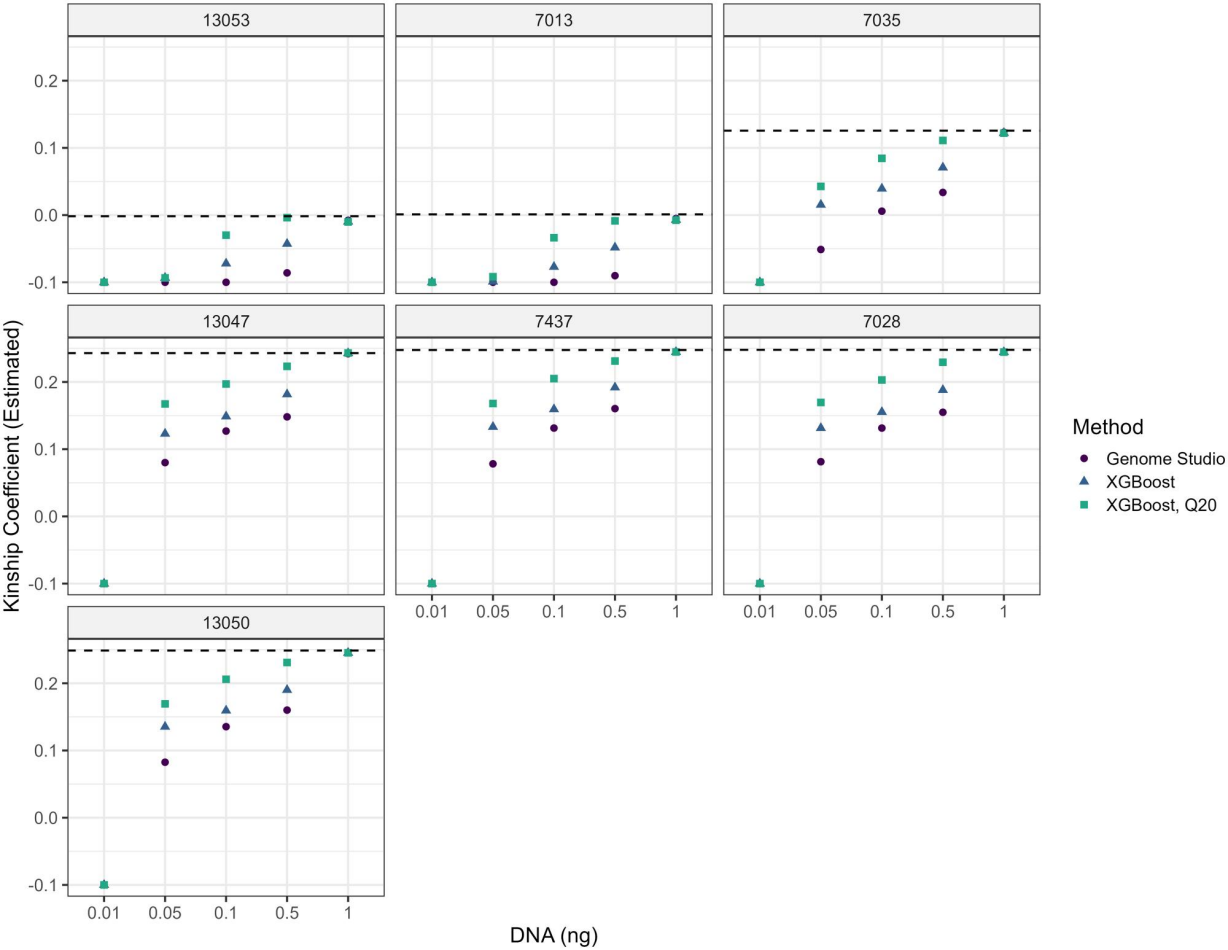
Kinship coefficient compared amongst the different methods at different DNA inputs. Relationships relative to NA7046 were analyzed (subpanels) and the kinship coefficient was estimated (y-axis) at various DNA input (x-axis) using both Genome Studio and XGBoost (with and without only considering a minimum genotype quality of 20, colors). The actual kinship coefficient is also depicted (dashed line, as estimated using 50 ng samples). Kinship coefficient estimates were clamped to −0.10 for presentation purposes.

The (true) kinship coefficient varies from 0 (unrelated individuals) to 0.50 (identity), with five relationship pairs having an expected coefficient of 0.25 (first-degree relatives) with an additional second-degree relative (7013; 0.125 is the expected value). In general, genotyping error reduces the estimated coefficient (Turner *et al.* 2022).The kinship coefficient was estimated considering both genotypes from GenomeStudio as well as using genotypes inferred from our model (Fig. 6, Table 24, available as supplementary data at *Bioinformatics Advances* online). While the 0.01 ng samples appear to harbor little signal of relatedness, 0.05 ng and higher titration levels capture hereditary signal. In addition, relatedness estimates better resemble their true values in both related and unrelated comparisons (see dashed lines, Fig. 6). This is also true of the second-degree relationship (7013, Fig. 6), though perhaps is better illustrated when there is at least 0.1 ng of starting material.

## 4 Discussion

### 4.1 Genotyping using supervised learning

SNP microarrays are used across a great many scientific disciplines. Because of their varied applications, generating reliable information across sample types is crucial to their use. GenomeStudio uses a proprietary unsupervised clustering method to assign genotypes. From a machine learning perspective, a supervised approach is more appropriate, especially as samples deviate from their clustered expectations.

Several classes of supervised methods were investigated, including linear (RMLR) and nonlinear (XGBoost and NN) approaches. XGBoost appears to provide both accurate genotypes as well as better class discrimination (e.g. F1 scores, Tables 6 and 7) than either RMLR or NN. While neural networks may be argued to be a superior technique, getting the most out of them often requires obscene quantities of finesse. In our studies, XGBoost performs better across DNA inputs (Tables 6 and 7), suggesting that it may generalize better to new samples (possibly of different quantity or quality). XGBoost also directly provides genotype probabilities. Many downstream genomic tools can operate on either raw genotype calls, or their corresponding likelihoods (Korneliussen *et al.* 2014, Nguyen *et al.* 2023). While these approaches have been limited to sequencing technologies, our approach may allow microarrays to be used in a similar fashion.

Genotype probabilities also have an immediate application. The GenCall is often used as the primary metric of SNP quality. Other measures, however, may be better suited to determine the quality of a call (Table 2). Genotype probabilities (as opposed to scores) provide a natural standard of quality, a measure that has (up until now) been absent from microarray technologies. Bear in mind that, depending on the machine learning method and intended use, some calibration may be required.

GenomeStudio shows good performance when data similar to those used to train it are considered (>1 ng). XGBoost, however, appears to generalize better across DNA inputs, suggesting a general benefit to the approach. Further improvements can also be seen with the application of a genotype quality filter using the Phred scaled score. The filter thresholds were set at 10, 20, and 30. As expected, more stringent thresholds led to higher accuracy. This trend is applicable at all input levels. The F1 scores of XGBoost of AB were notably better than GenomeStudio, indicating better performance of XGboost over GenomeStudio when classifying heterozygotes. The final model of XGBoost also showed the same improvements over GenomeStudio in both F1 score and accuracy. The results of the model trained on the modified five individual training set showed similar F1 and accuracy scores, suggesting the relationships of the individuals did not influence the results from the model (Tables 7–10 and 16–23, available as supplementary data at *Bioinformatics Advances* online). The addition of a concordance filter showed minimal improvement. The AB F1 score showed a decrease in model performance at the lowest DNA input. This could be since the filter only keeps SNP calls that are incorrectly classified by GenomeStudio. The F1 scores for homozygous AA, and BB showed a small amount of improvement. Overall, reduced genotyping error has led to improvements in kinship coefficient estimation.

## 5 Conclusion

The three machine learning models were based on easily accessible packages, Keras, XGBoost and sci-kit learn. The first step of the study focused on determining which model is most suitable, with XGBoost showing the most promise. We then trained XGBoost on the full dataset, which showed improved performance over Genomestudio with the addition of two filters: a concordance filter and a Phred quality filter. By increasing the Phred filter and adding a concordance filter, XGBoost achieved 98% accuracy on a 0.05 ng sample. Future directions could involve using poor quality samples (such as UV degradation) with these models. Additionally, kinship identification is another area of exploration as genotyping error adversely affects the accuracy of relationship identification (de Vries *et al.* 2022, Turner *et al.* 2022), often in unintuitive ways. It is also worth investigating other technologies: since the classifier was only trained on Illumina’s 5-4 omni chip, further study will be required to determine whether these results may generalize to other SNP microarrays.

## Supplementary Material

vbag086_Supplementary_Data

## Data Availability

The source code for the ML models are available at https://github.com/RiotPumpkin/SNP-microarray-machine-learning-models. A script for calibrated XGBoost probabilities using platt scaling is included. The cell lines were obtained from NIGMS Human Genetic Cell Repository at the Coriell Institute for Medical Research and are commercially available for research use. Donors consent to having their cell lines used in research studies and publications by qualified researchers affiliated with accredited scientific, medical, educational, or industrial institutions. The study did not involve collecting new data from human subjects.
