# Supplementary material for: Machine learning improves SNP microarray performance in challenged samples
Source: Bioinform Adv. 2026 Mar 21;6(1):vbag086. doi: 10.1093/bioadv/vbag086 (PMC13091614; doi:10.1093/bioadv/vbag086)
Supplement: vbag086_Supplementary_Data [file vbag086_supplementary_data.docx]

**Supplemental material**


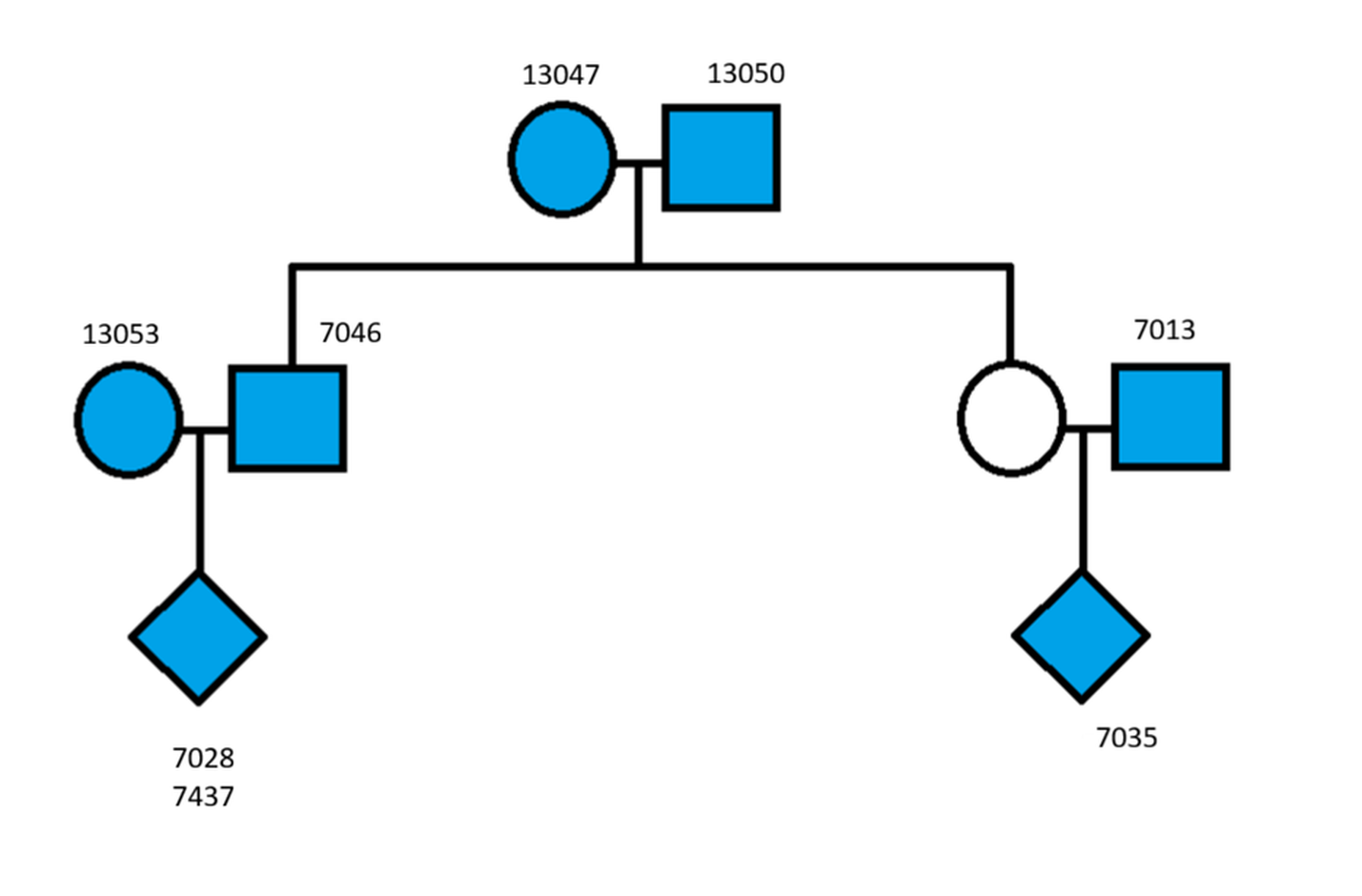


Supplemental Figure 1. The pedigree of the individuals used in this study, with blue-shaded shapes indicating the individuals included in the study. In total there are eight individuals.

Supplemental Table 1. Individuals used in the testing and training set for training the full model.

| Training | 7013, 7035, 13050, 7437, 13047, 7028, 13053 |
| --- | --- |
| Testing | 7046 |
| Female | 10347, 13053 |
| Male | 7046, 13050 |

Supplemental Table 2. Individuals used in the testing and training set. The individual 7013 had no familial relationship except with 7035. 7013 and 7035 were chosen to be in the test set to prevent and to test for any familial data leakage.

| Training | 7046, 7437, 13047, 7028, 13053, 13050 |
| --- | --- |
| Testing | 7013, 7035 |
| Female | 10347, 13053 |
| Male | 7046, 13050 |

Supplemental Table 3. Accuracy achieved by each classifier at different Phred quality filter. The percentage point difference in the parenthesis measures the difference between classifier and Genomestudio. Positive values indicate better performance

| DNA input | Phred qual | RMLR | XGBoost | NN |
| --- | --- | --- | --- | --- |
| 1 ng | 0 | 93.99%(-5.94%) | 99.92%(-0.01%) | 98.44%(-1.49%) |
|  | 10 | 96.64%(-3.31%) | 99.94%(-0.01%) | 99.06%(-0.89%) |
|  | 20 | 99.07%(-0.88%) | 99.97%(+0.02%) | 99.68%(-0.28%) |
|  | 30 | 99.64%(-0.33%) | 99.98%(+0.01%) | 99.82%(-0.15%) |
| 0.5 ng | 0 | 93.99%(-2.46%) | 96.75%(+0.30%) | 95.68%(-0.77%) |
|  | 10 | 96.64%(-0.48%) | 97.79%(+0.68%) | 97.33%(+0.22%) |
|  | 20 | 99.07%(+1.29%) | 99.21%(+1.43%) | 98.84%(+1.06%) |
|  | 30 | 99.64%(+1.34%) | 99.71%(+1.41%) | 99.46%(+1.16%) |
| 0.1 ng | 0 | 90.92%(-4.73%) | 97.00%(+1.35%) | 94.33%(-1.33%) |
|  | 10 | 95.75%(-0.60%) | 97.86%(+1.52%) | 96.45%(+0.11%) |
|  | 20 | 99.10%(+2.11%) | 98.99%(+1.99%) | 98.43%(+1.44%) |
|  | 30 | 99.68%(+2.25%) | 99.46%(+2.04%) | 99.26%(+1.83%) |
| 0.05 ng | 0 | 90.08%(-3.86%) | 95.06%(+1.12%) | 93.94%(+0.00%) |
|  | 10 | 94.64%(-0.16%) | 96.11%(+1.31%) | 96.12%(+1.33%) |
|  | 20 | 98.30%(+2.76%) | 97.74%(+2.20%) | 98.20%(+2.66%) |
|  | 30 | 99.32%(+3.61%) | 98.59%(+2.88%) | 99.25%(+3.54%) |
| 0.01 ng | 0 | 61.39%(-6.59%) | 68.29%(+0.30%) | 69.19%(+1.20%) |
|  | 10 | 69.57%(-4.04%) | 73.18%(-0.42%) | 76.01%(+2.40%) |
|  | 20 | 90.84%(+12.52%) | 86.78%(+8.46%) | 93.87%(+15.55%) |
|  | 30 | 89.26%(+14.50%) | 97.71%(+22.95%) | 98.86%(+24.10%) |

Supplemental Table 4. The F1 Score for the AB class of different classifiers at different DNA input and Phred quality scores. The percentage point difference in the parenthesis measures the difference between classifier and Genomestudio. Positive values indicate better performance

| DNA input | Phred qual | RMLR F1 AB | XGBoost F1 AB | NN F1 AB |  |
| --- | --- | --- | --- | --- | --- |
| 1 ng | 0 | 76.06% (-23.71%) | 99.72% (-0.05%) | 94.43% (-5.34%) | |
|  | 10 | 83.68% (-16.15%) | 99.80% (-0.03%) | 96.45% (-3.39%) | |
|  | 20 | 93.56% (-6.30%) | 99.91% (+0.05%) | 98.61% (-1.25%) | |
|  | 30 | 97.00% (-2.91%) | 99.95% (+0.04%) | 99.12% (-0.78%) | |
| 0.5 ng | 0 | 70.26% (-16.50%) | 88.62% (+1.87%) | 83.51% (-3.24%) | |
|  | 10 | 83.77% (-5.02%) | 91.83% (+3.04%) | 88.72% (-0.07%) | |
|  | 20 | 96.29% (+4.72%) | 96.76% (+5.19%) | 94.48% (+2.91%) | |
|  | 30 | 98.67% (+4.79%) | 98.64% (+4.76%) | 97.05% (+3.16%) | |
| 0.1 ng | 0 | 67.78% (-15.01%) | 88.45% (+5.67%) | 77.53% (-5.25%) | |
|  | 10 | 81.14% (-3.52%) | 91.29% (+6.63%) | 84.07% (-0.58%) | |
|  | 20 | 94.13% (+6.54%) | 95.41% (+7.82%) | 91.80% (+4.21%) | |
|  | 30 | 97.75% (+7.81%) | 97.26% (+7.31%) | 95.38% (+5.44%) | |
| 0.05 ng | 0 | 63.59% (-10.88%) | 80.12% (+5.65%) | 74.34% (-0.13%) | |
|  | 10 | 76.13% (-0.28%) | 83.35% (+6.94%) | 90.91% (+14.50%) | |
|  | 20 | 91.47% (+10.98%) | 89.11% (+8.62%) | 89.45% (+8.96%) | |
|  | 30 | 96.56% (+13.74%) | 92.15% (+9.33%) | 94.89% (+12.07%) | |
| 0.01 ng | 0 | 36.59% (+12.56%) | 41.76% (+17.73%) | 42.06% (+18.03%) | |
|  | 10 | 42.15% (+21.33%) | 46.90% (+26.08%) | 48.21% (+27.39%) | |
|  | 20 | 58.99% (+42.61%) | 63.18% (+46.80%) | 62.73% (+46.35%) | |
|  | 30 | 78.40% (+65.55%) | 76.15% (+63.30%) | 70.80% (+57.95%) | |

Supplemental Table 5. The F1 Score for the AA class of different classifiers at different DNA input and Phred quality scores. The percentage point difference in the parenthesis measures the difference between classifier and Genomestudio. Positive values indicate better performance

| DNA input | Phred qual | RMLR F1 AA | XGBoost F1 AA | NN F1 AA |
| --- | --- | --- | --- | --- |
| 1 ng | 0 | 96.75% (+3.23%) | 99.98% (+0.01%) | 99.17% (+0.81%) |
|  | 10 | 98.20% (+1.79%) | 99.98% (+0.00%) | 99.47% (+0.51%) |
|  | 20 | 99.45% (+0.54%) | 99.99% (+0.00%) | 99.76% (+0.22%) |
|  | 30 | 99.78% (+0.21%) | 99.99% (+0.00%) | 99.80% (+0.19%) |
| 0.5 ng | 0 | 97.20% (+0.52%) | 98.77% (-1.05%) | 97.71% (+0.02%) |
|  | 10 | 98.88% (-0.53%) | 99.25% (-0.91%) | 99.03% (-0.69%) |
|  | 20 | 99.68% (-0.81%) | 99.71% (-0.84%) | 99.74% (-0.87%) |
|  | 30 | 99.84% (-0.79%) | 99.87% (-0.82%) | 99.85% (-0.80%) |
| 0.1 ng | 0 | 92.45% (+5.64%) | 97.55% (+0.54%) | 97.31% (+0.78%) |
|  | 10 | 96.39% (+1.94%) | 98.29% (+0.03%) | 98.09% (+0.23%) |
|  | 20 | 99.31% (-0.74%) | 99.41% (-0.84%) | 99.10% (-0.53%) |
|  | 30 | 99.79% (-0.82%) | 99.81% (-0.84%) | 99.59% (-0.62%) |
| 0.05 ng | 0 | 96.12% (+0.15%) | 97.73% (-1.46%) | 96.52% (-0.25%) |
|  | 10 | 98.06% (-1.21%) | 98.20% (-1.34%) | 98.45% (-1.60%) |
|  | 20 | 99.43% (-2.07%) | 98.82% (-1.46%) | 99.52% (-2.16%) |
|  | 30 | 99.76% (-2.44%) | 99.17% (-1.86%) | 99.78% (-2.46%) |
| 0.01 ng | 0 | 72.69% (+7.26%) | 74.20% (+5.75%) | 75.38% (+4.57%) |
|  | 10 | 82.93% (+1.94%) | 79.01% (+5.86%) | 84.36% (+0.50%) |
|  | 20 | 97.45% (-16.73%) | 90.92% (-10.20%) | 98.34% (-17.62%) |
|  | 30 | 95.09% (-15.48%) | 98.58% (-18.97%) | 99.90% (-20.29%) |

Supplemental Table 6. The F1 Score for the BB class of different classifiers at different DNA input and Phred quality scores. The percentage point difference in the parenthesis measures the difference between classifier and Genomestudio. Positive values indicate better performance

| DNA input | Phred qual | RMLR F1 BB | XGBoost F1 BB | NN F1 BB |
| --- | --- | --- | --- | --- |
| 1 ng | 0 | 96.35% (+3.59%) | 99.93% (+0.01%) | 99.03% (+0.90%) |
|  | 10 | 98.04% (+1.91%) | 99.95% (+0.01%) | 99.44% (+0.51%) |
|  | 20 | 99.53% (+0.42%) | 99.97% (-0.02%) | 99.85% (+0.11%) |
|  | 30 | 99.84% (+0.14%) | 99.99% (-0.01%) | 99.94% (+0.03%) |
| 0.5 ng | 0 | 92.45% (+5.64%) | 97.55% (+0.54%) | 97.31% (+0.78%) |
|  | 10 | 96.39% (+1.94%) | 98.29% (+0.03%) | 98.09% (+0.23%) |
|  | 20 | 99.31% (-0.74%) | 99.41% (-0.84%) | 99.10% (-0.53%) |
|  | 30 | 99.79% (-0.82%) | 99.81% (-0.84%) | 99.59% (-0.62%) |
| 0.1 ng | 0 | 92.58% (+5.06%) | 98.00% (-0.36%) | 96.78% (+0.86%) |
|  | 10 | 96.25% (+1.67%) | 98.58% (-0.66%) | 97.63% (+0.29%) |
|  | 20 | 98.96% (-0.81%) | 99.36% (-1.21%) | 98.80% (-0.65%) |
|  | 30 | 99.63% (-1.12%) | 99.68% (-1.17%) | 99.43% (-0.92%) |
| 0.05 ng | 0 | 92.28% (+4.41%) | 96.66% (+0.03%) | 93.94% (+2.75%) |
|  | 10 | 95.89% (+1.32%) | 97.42% (-0.21%) | 96.12% (+1.09%) |
|  | 20 | 98.70% (-1.12%) | 98.65% (-1.07%) | 98.20% (-0.62%) |
|  | 30 | 99.47% (-1.69%) | 99.25% (-1.47%) | 99.25% (-1.47%) |
| 0.01 ng | 0 | 66.75% (+14.03%) | 77.68% (+3.11%) | 75.82% (+4.96%) |
|  | 10 | 73.02% (+11.72%) | 82.19% (+2.55%) | 80.30% (+4.44%) |
|  | 20 | 91.30% (-10.82%) | 92.16% (-11.68%) | 94.44% (-13.96%) |
|  | 30 | 90.41% (-11.70%) | 98.87% (-20.16%) | 89.53% (-10.82%) |
|  |  |  |  |  |

Supplemental Table 7. The accuracy for XGBoost based on the full training data at different DNA input and Phred quality scores. Accuracy is reported with and without a concordance at different DNA inputs and phred quality score filter. The percentage point difference in the parenthesis measures the difference between classifier and Genomestudio. Positive values indicate better performance

| DNA input | Phred Filter | Accuracy Without concordance | Accuracy With concordance |
| --- | --- | --- | --- |
| 1 ng | 0 | 99.94% (+0.01%) | 99.95% (+0.02%) |
|  | 10 | 99.95% (+0.02%) | 99.95% (+0.02%) |
|  | 20 | 99.96% (+0.03%) | 99.96% (+0.03%) |
|  | 30 | 99.97% (+0.04%) | 99.97% (+0.04%) |
| 0.5 ng | 0 | 98.47% (+1.80%) | 98.90% (+2.23%) |
|  | 10 | 98.92% (+2.10%) | 99.17% (+2.35%) |
|  | 20 | 99.56% (+2.57%) | 99.62% (+2.63%) |
|  | 30 | 99.82% (+2.69%) | 99.84% (+2.71%) |
| 0.1 ng | 0 | 98.01% (+2.13%) | 98.32% (+2.44%) |
|  | 10 | 98.45% (+2.44%) | 98.64% (+2.63%) |
|  | 20 | 99.09% (+2.91%) | 99.15% (+2.97%) |
|  | 30 | 99.42% (+3.10%) | 99.44% (+3.12%) |
| 0.05 ng | 0 | 96.05% (+1.85%) | 96.65% (+2.44%) |
|  | 10 | 96.71% (+2.32%) | 97.09% (+2.70%) |
|  | 20 | 97.71% (+3.04%) | 97.85% (+3.17%) |
|  | 30 | 98.31% (+3.40%) | 98.36% (+3.44%) |
| 0.01 ng | 0 | 79.52% (+5.72%) | 86.96% (+13.16%) |
|  | 10 | 90.24% (+14.23%) | 94.31% (+18.30%) |
|  | 20 | 98.74% (+25.69%) | 99.04% (+25.99%) |
|  | 30 | 99.74% (+28.43%) | 99.76% (+28.45%) |

Supplemental 8. The F1 Score for the AB class of XGBoost based on full training data at different DNA input and Phred quality scores. The percentage point difference in the parenthesis measures the difference between classifier and Genomestudio. Positive values indicate better performance

| DNA input | Phred Filter | AB F1 Without concordance | AB F1 With concordance |
| --- | --- | --- | --- |
| 1 ng | 0 | 99.82% (+0.05%) | 99.82% (+0.05%) |
|  | 10 | 99.84% (+0.07%) | 99.84% (+0.07%) |
|  | 20 | 99.88% (+0.11%) | 99.88% (+0.11%) |
|  | 30 | 99.91% (+0.14%) | 99.91% (+0.14%) |
| 0.5 ng | 0 | 94.55% (+7.18%) | 95.62% (+8.25%) |
|  | 10 | 96.09% (+8.32%) | 96.66% (+8.90%) |
|  | 20 | 98.37% (+10.04%) | 98.46% (+10.13%) |
|  | 30 | 99.31% (+10.53%) | 99.34% (+10.55%) |
| 0.1 ng | 0 | 92.40% (+9.08%) | 92.78% (+9.46%) |
|  | 10 | 93.93% (+10.33%) | 94.06% (+10.46%) |
|  | 20 | 96.27% (+12.21%) | 96.15% (+12.09%) |
|  | 30 | 97.52% (+13.05%) | 97.39% (+12.92%) |
| 0.05 ng | 0 | 84.05% (+9.10%) | 84.34% (+9.39%) |
|  | 10 | 86.20% (+10.93%) | 86.06% (+10.79%) |
|  | 20 | 89.76% (+13.81%) | 89.16% (+13.21%) |
|  | 30 | 92.01% (+15.38%) | 91.37% (+14.74%) |
| 0.01 ng | 0 | 50.23% (+27.94%) | 38.92% (+16.63%) |
|  | 10 | 58.59% (+38.74%) | 44.12% (+24.27%) |
|  | 20 | 78.30% (+60.16%) | 75.11% (+56.97%) |
|  | 30 | 92.93% (+75.45%) | 92.39% (+74.91%) |

Supplemental Table 9. The F1 Score for the AA class of XGBoost based on full training data at different DNA input and Phred quality scores. The percentage point difference in the parenthesis measures the difference between classifier and Genomestudio. Positive values indicate better performance

| DNA input | Phred Filter | AA F1 Without concordance | AA F1 With concordance |
| --- | --- | --- | --- |
| 1 ng | 0 | 99.94% (-0.05%) | 99.95% (-0.01%) |
|  | 10 | 99.94% (-0.05%) | 99.96% (-0.01%) |
|  | 20 | 99.94% (-0.05%) | 99.97% (+0.01%) |
|  | 30 | 99.94% (-0.05%) | 99.98% (+0.01%) |
| 0.5 ng | 0 | 99.52% (+1.59%) | 98.78% (+0.61%) |
|  | 10 | 99.65% (+1.60%) | 99.16% (+0.91%) |
|  | 20 | 99.81% (+1.60%) | 99.69% (+1.38%) |
|  | 30 | 99.89% (+1.55%) | 99.89% (+1.54%) |
| 0.1 ng | 0 | 99.02% (+1.54%) | 98.69% (+0.94%) |
|  | 10 | 99.19% (+1.60%) | 99.03% (+1.22%) |
|  | 20 | 99.45% (+1.72%) | 99.49% (+1.60%) |
|  | 30 | 99.62% (+1.76%) | 99.70% (+1.76%) |
| 0.05 ng | 0 | 97.98% (+1.53%) | 97.49% (+0.63%) |
|  | 10 | 98.23% (+1.65%) | 98.01% (+1.02%) |
|  | 20 | 98.62% (+1.87%) | 98.76% (+1.59%) |
|  | 30 | 98.89% (+1.99%) | 99.17% (+1.86%) |
| 0.01 ng | 0 | 85.86% (+3.22%) | 85.70% (+2.72%) |
|  | 10 | 93.82% (+8.23%) | 94.00% (+9.30%) |
|  | 20 | 99.17% (+15.49%) | 99.29% (+16.34%) |
|  | 30 | 99.83% (+17.19%) | 99.86% (+18.22%) |

Supplemental Table 10. The F1 Score for the BB class of XGBoost based on full training data. The percentage point difference in the parenthesis measures the difference between classifier and Genomestudio. Positive values indicate better performance

| DNA input | Phred Filter | BB F1 Without concordance | BB F1 With concordance |
| --- | --- | --- | --- |
| 1 ng | 0 | 99.95% (-0.01%) | 99.95% (-0.01%) |
|  | 10 | 99.96% (-0.01%) | 99.96% (-0.00%) |
|  | 20 | 99.97% (+0.01%) | 99.97% (+0.01%) |
|  | 30 | 99.98% (+0.01%) | 99.98% (+0.01%) |
| 0.5 ng | 0 | 98.78% (+0.61%) | 99.11% (+0.93%) |
|  | 10 | 99.16% (+0.91%) | 99.34% (+1.09%) |
|  | 20 | 99.69% (+1.38%) | 99.72% (+1.41%) |
|  | 30 | 99.89% (+1.54%) | 99.89% (+1.55%) |
| 0.1 ng | 0 | 98.69% (+0.94%) | 98.90% (+1.16%) |
|  | 10 | 99.03% (+1.22%) | 99.15% (+1.34%) |
|  | 20 | 99.49% (+1.60%) | 99.52% (+1.63%) |
|  | 30 | 99.70% (+1.76%) | 99.70% (+1.77%) |
| 0.05 ng | 0 | 97.49% (+0.63%) | 97.95% (+1.09%) |
|  | 10 | 98.01% (+1.02%) | 98.29% (+1.30%) |
|  | 20 | 98.76% (+1.59%) | 98.84% (+1.68%) |
|  | 30 | 99.17% (+1.86%) | 99.19% (+1.87%) |
| 0.01 ng | 0 | 85.70% (+2.72%) | 91.85% (+8.87%) |
|  | 10 | 94.00% (+9.30%) | 96.76% (+12.06%) |
|  | 20 | 99.29% (+16.34%) | 99.47% (+16.52%) |
|  | 30 | 99.86% (+18.22%) | 99.86% (+18.22%) |

Supplemental Table 11. Relative call rates for each of the classifier at different DNA input and phred quality filter. The percentage and raw count is reported under each respective classifier.

| DNA input | Total SNPs | Phred | RMLR | XGBoost | NN |
| --- | --- | --- | --- | --- | --- |
| 1 ng | 4,120,603 | 10 | 3,896,745 | 4,118,403 | 4052904 |
|  |  |  | 94.57% | 99.95% | 98.36% |
|  |  | 20 | 3,463,083 | 4,111,654 | 3805167 |
|  |  |  | 84.04% | 99.78% | 92.34% |
|  |  | 30 | 2,838,823 | 4,099,647 | 3097507 |
|  |  |  | 68.89% | 99.49% | 75.17% |
| 0.5 ng | 4081492 | 10 | 3577828 | 3987230 | 3900187 |
|  |  |  | 87.66% | 97.69% | 95.56% |
|  |  | 20 | 2769805 | 3795919 | 3231048 |
|  |  |  | 67.86% | 93.00% | 79.16% |
|  |  | 30 | 2241071 | 3587165 | 2548239 |
|  |  |  | 54.91% | 87.89% | 62.43% |
| 0.1 ng | 4036424 | 10 | 3519292 | 3958056 | 3801705 |
|  |  |  | 87.19% | 98.06% | 94.18% |
|  |  | 20 | 2675430 | 3787694 | 3073746 |
|  |  |  | 66.28% | 93.84% | 76.15% |
|  |  | 30 | 2133385 | 3602838 | 2462222 |
|  |  |  | 52.85% | 89.26% | 61.00% |
| 0.05 ng | 3973861 | 10 | 3439880 | 3879574 | 3730293 |
|  |  |  | 86.56% | 97.63% | 93.87% |
|  |  | 20 | 2466536 | 3658091 | 2945246 |
|  |  |  | 62.07% | 92.05% | 74.12% |
|  |  | 30 | 1893598 | 3409343 | 2258556 |
|  |  |  | 47.65% | 85.79% | 56.84% |
| 0.01 ng | 2195952 | 10 | 1483775 | 1863947 | 1601165 |
|  |  |  | 67.57% | 84.88% | 72.91% |
|  |  | 20 | 490578 | 1263107 | 787557 |
|  |  |  | 22.34% | 57.52% | 35.86% |
|  |  | 30 | 85851 | 876843 | 325503 |
|  |  |  | 3.91% | 39.93% | 14.82% |

Supplemental Table 12 Absolute call rates for each of the classifier at different DNA input and phred quality filter. The percentage and raw count is reported under each respective classifier.

| DNA input | Total SNPs | Phred | RMLR | XGBoost | NN |
| --- | --- | --- | --- | --- | --- |
| 1 ng | 4198873 | 10 | 3,896,745 | 4,118,403 | 4052904 |
|  |  |  | 92.80% | 98.08% | 96.52% |
|  |  | 20 | 3,463,083 | 4,111,654 | 3805167 |
|  |  |  | 82.48% | 97.92% | 90.62% |
|  |  | 30 | 2,838,823 | 4,099,647 | 3097507 |
|  |  |  | 67.61% | 97.64% | 73.77% |
| 0.5 ng |  | 10 | 3577828 | 3987230 | 3900187 |
|  |  |  | 85.21% | 94.96% | 92.89% |
|  |  | 20 | 2769805 | 3795919 | 3231048 |
|  |  |  | 65.97% | 90.40% | 76.95% |
|  |  | 30 | 2241071 | 3587165 | 2548239 |
|  |  |  | 53.37% | 85.43% | 60.69% |
| 0.1 ng |  | 10 | 3519292 | 3958056 | 3801705 |
|  |  |  | 83.82% | 94.26% | 90.54% |
|  |  | 20 | 2675430 | 3787694 | 3073746 |
|  |  |  | 63.72% | 90.21% | 73.20% |
|  |  | 30 | 2133385 | 3602838 | 2462222 |
|  |  |  | 50.81% | 85.80% | 58.64% |
| 0.05 ng |  | 10 | 3439880 | 3879574 | 3730293 |
|  |  |  | 81.92% | 92.40% | 88.84% |
|  |  | 20 | 2466536 | 3658091 | 2945246 |
|  |  |  | 58.74% | 87.12% | 70.14% |
|  |  | 30 | 1893598 | 3409343 | 2258556 |
|  |  |  | 45.10% | 81.20% | 53.79% |
| 0.01 ng |  | 10 | 1483775 | 1863947 | 1601165 |
|  |  |  | 35.34% | 44.39% | 38.13% |
|  |  | 20 | 490578 | 1263107 | 787557 |
|  |  |  | 11.68% | 30.08% | 18.76% |
|  |  | 30 | 85851 | 876843 | 325503 |
|  |  |  | 2.04% | 20.88% | 7.75% |

Supplmental Table 13. The relative call rate for XGBoost trained on the full training data. The raw call rate and percentage is calculated for each phred quality filter without and with a concordance filter.

| Total SNPs | 30 Phred W/O Con | 20 Phred W/O | 10 Phred W/O | Total SNPs | 30 phred W | 20 Phred W | 10 Phred W | DNA input |
| --- | --- | --- | --- | --- | --- | --- | --- | --- |
| 4120603 | 4117849 | 4118912 | 4120060 | 4119877 | 4117841 | 4118846 | 4119600 | 1 ng |
|  | 99.93% | 99.96% | 99.99% |  | 99.95% | 99.97% | 99.99% |  |
|  |  |  |  |  |  |  |  |  |
| 4081492 | 3864006 | 3947693 | 4041045 | 3962650 | 3813397 | 3878925 | 3940451 | 0.5 ng |
|  | 94.67% | 96.72% | 99.01% |  | 96.23% | 97.89% | 99.44% |  |
|  |  |  |  |  |  |  |  |  |
| 4036424 | 3833510 | 3914953 | 3998926 | 3913490 | 3784068 | 3844489 | 3893587 | 0.1 ng |
|  | 94.97% | 96.99% | 99.07% |  | 96.69% | 98.24% | 99.49% |  |
|  |  |  |  |  |  |  |  |  |
| 3973861 | 3692600 | 3801230 | 3916609 | 3835959 | 3646262 | 3732980 | 3805982 | 0.05 ng |
|  | 92.92% | 95.66% | 98.56% |  | 95.05% | 97.32% | 99.22% |  |
|  |  |  |  |  |  |  |  |  |
| 2195952 | 935386 | 1097589 | 1624856 | 1612535 | 927612 | 1051521 | 1358010 | 0.01 ng |
|  | 42.60% | 49.98% | 73.99% |  | 57.53% | 65.21% | 84.22% |  |

Supplemental Table 14. Absolute call rates for XGBoost trained on the full training data. The raw call rate and percentage is calculated for each phred quality filter without and with a concordance filter.

| Total SNPs | 30 Phred W/O Con | 20 Phred W/O | 10 Phred W/O | Total SNPs | 30 phred W | 20 Phred W | 10 Phred W | DNA input |
| --- | --- | --- | --- | --- | --- | --- | --- | --- |
| 4198873 | 4117849 | 4118912 | 4120060 | 4119877 | 4117841 | 4118846 | 4119600 | 1 ng |
|  | 98.07% | 98.10% | 98.12% |  | 98.07% | 98.09% | 98.11% |  |
|  |  |  |  |  |  |  |  |  |
|  | 3864006 | 3947693 | 4041045 | 3962650 | 3813397 | 3878925 | 3940451 | 0.5 ng |
|  | 92.02% | 94.02% | 96.24% |  | 90.82% | 92.38% | 93.85% |  |
|  |  |  |  |  |  |  |  |  |
|  | 3833510 | 3914953 | 3998926 | 3913490 | 3784068 | 3844489 | 3893587 | 0.1 ng |
|  | 91.30% | 93.24% | 95.24% |  | 90.12% | 91.56% | 92.73% |  |
|  |  |  |  |  |  |  |  |  |
|  | 3692600 | 3801230 | 3916609 | 3835959 | 3646262 | 3732980 | 3805982 | 0.05 ng |
|  | 87.94% | 90.53% | 93.28% |  | 86.84% | 88.90% | 90.64% |  |
|  |  |  |  |  |  |  |  |  |
|  | 935386 | 1097589 | 1624856 | 1612535 | 927612 | 1051521 | 1358010 | 0.01 ng |
|  | 22.28% | 26.14% | 38.70% |  | 22.09% | 25.04% | 32.34% |  |


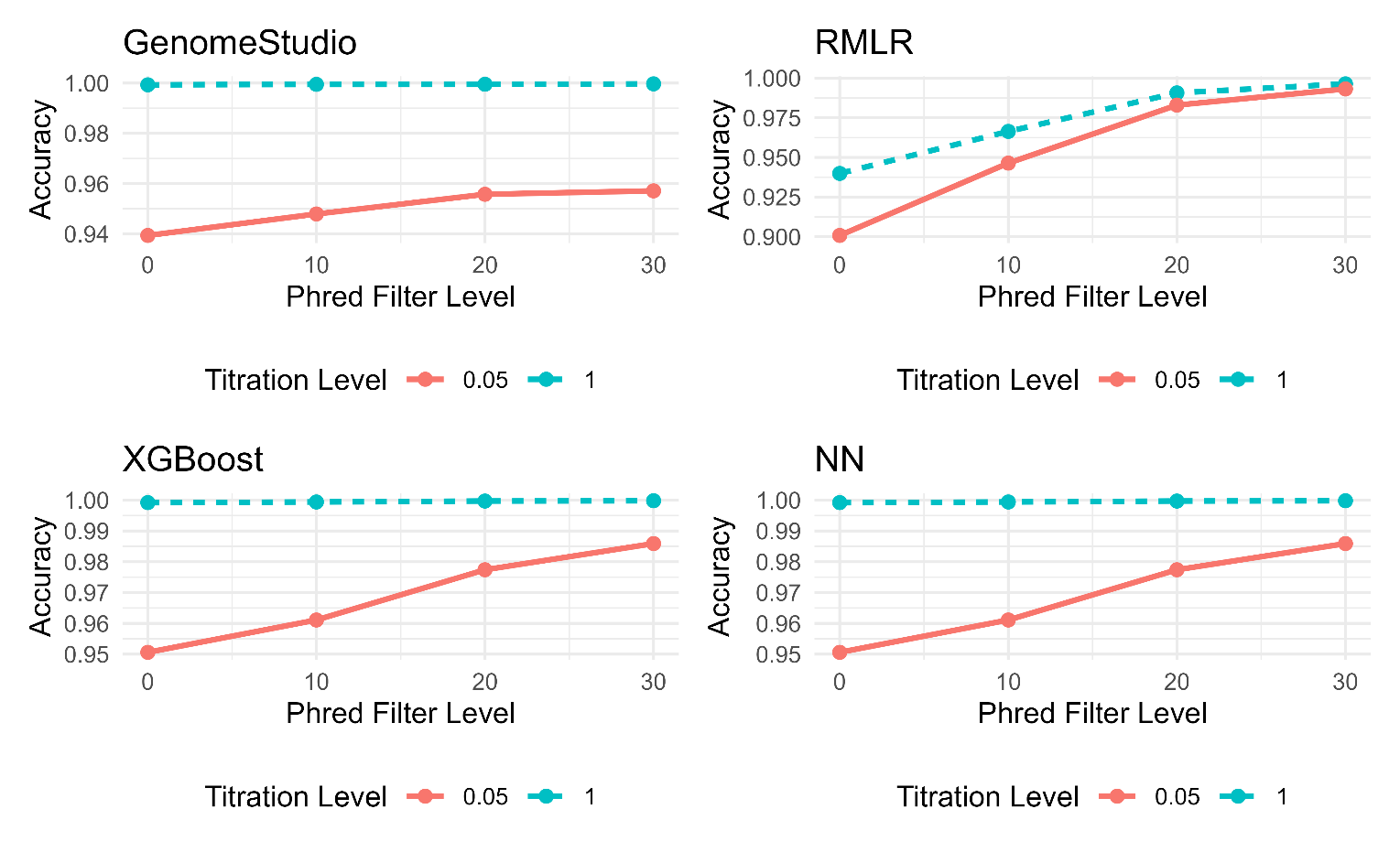


Supplemental Figure 2. The accuracy of each class of classifiers at each titration level (Ng) and Phred quality score.


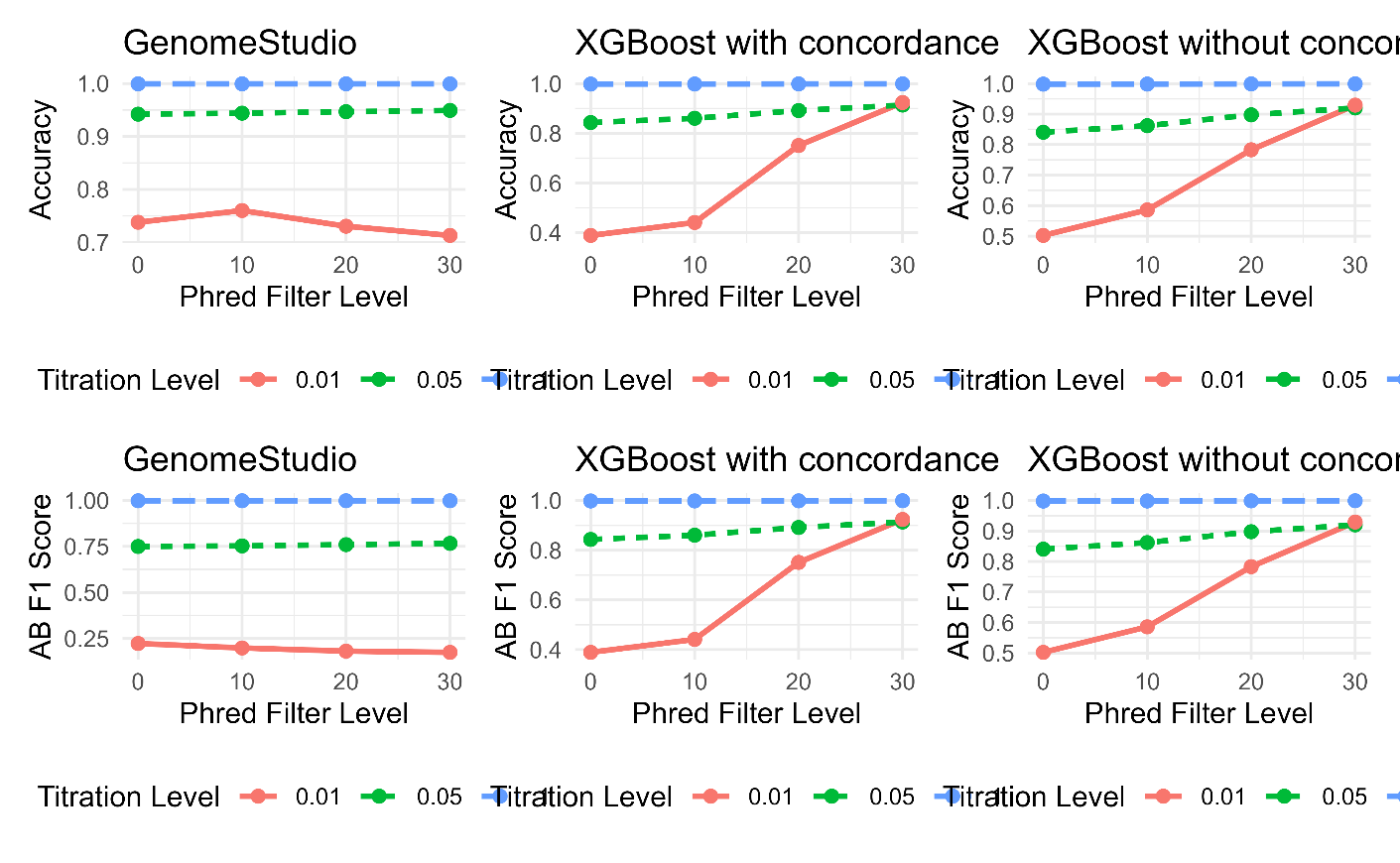


Supplemental Figure 3. Accuracy and AB F1 score trends for XGBoost and Genomestudio at different DNA inputs (Ng) and Phred quality score.


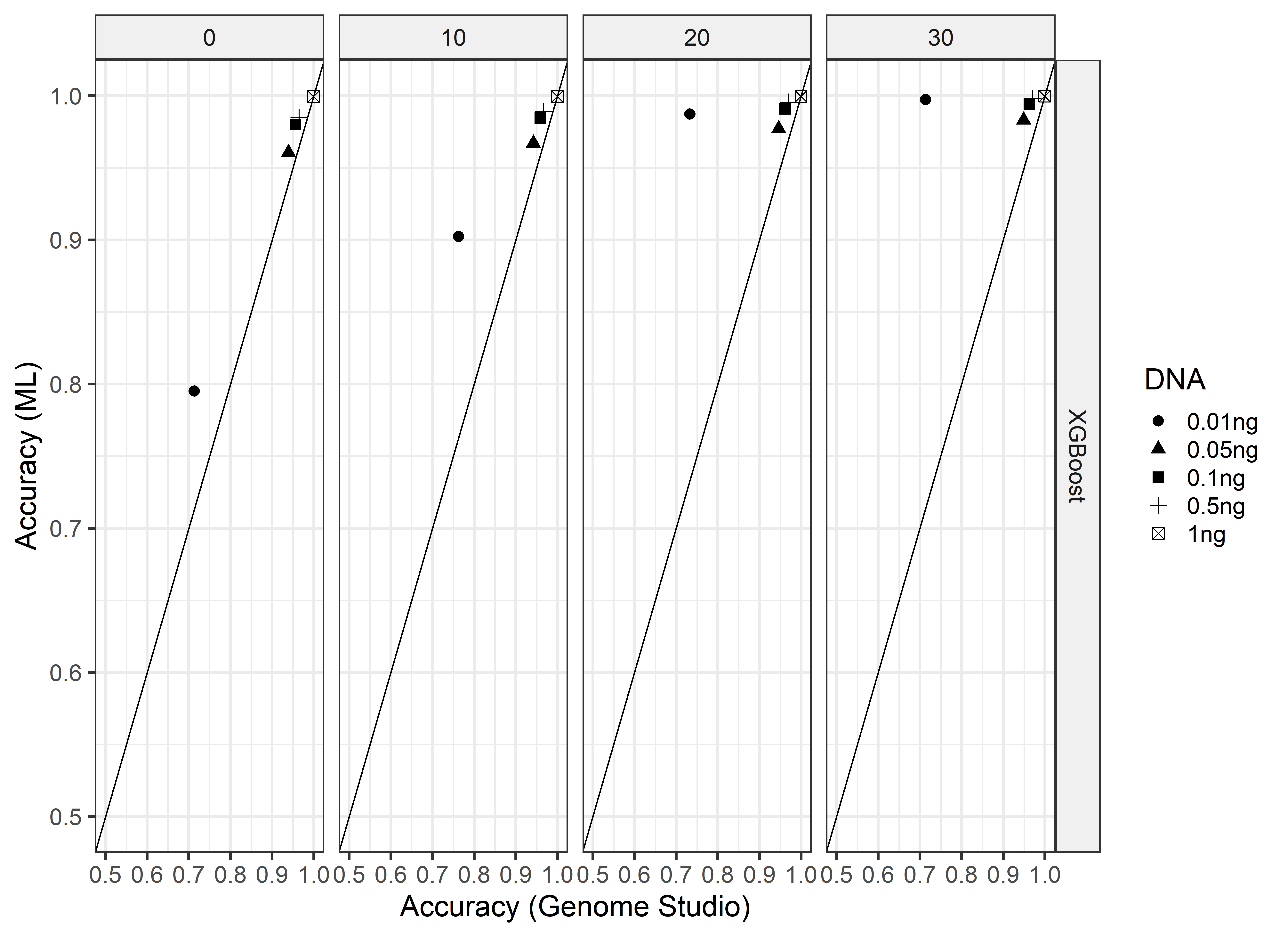


Supplemental Figure 4. Accuracy of XGBoost at different phred quality score and different dilution levels in comparison to GenomeStudio. The diagonal line indicates same performance between XGBoost and GenomeStudio. At dilution levels lower than 1ng XGBoost performs better.

Supplemental Table 15. Call rate of GenomeStudio with a total of 4198873 SNPs. Column header contains sample ID and the mean call rate across all samples.

| DNA input (ng) | 7046 | 7437 | 7028 | 13053 | 7013 | 7035 | 13047 | 13050 | Mean call rate |
| --- | --- | --- | --- | --- | --- | --- | --- | --- | --- |
| 50 | 99.64% | 99.05% | 99.41% | 99.56% | 99.73% | 99.39% | 99.51% | 99.53% | 99.48% |
| 1 | 98.28% | 97.93% | 98.43% | 98.61% | 98.54% | 98.29% | 98.61% | 98.62% | 98.41% |
| 0.5 | 97.46% | 96.98% | 97.70% | 97.87% | 97.73% | 97.41% | 97.72% | 95.78% | 97.33% |
| 0.1 | 96.38% | 93.23% | 95.44% | 96.47% | 96.40% | 90.61% | 96.95% | 93.31% | 94.85% |
| 0.05 | 94.90% | 94.30% | 95.82% | 95.59% | 95.31% | 94.91% | 96.32% | 95.96% | 95.39% |
| 0.01 | 52.47% | 74.81% | 88.25% | 88.76% | 85.41% | 55.44% | 92.36% | 90.14% | 78.46% |

Additionally, the final model was also retrained on a modified training set of 5 individuals with individual, 7013, (supplemental table 2) retained for testing purposes. The 5 individuals in the training set were chosen to have no relationship with 7013 to weed out familial data leakage.

Supplemental Table 16. NA7013 F1 Score for the AA class of XGBoost based on 5 individuals in the training set at different DNA input and Phred quality scores. The percentage point difference in the parenthesis measures the difference between classifier and Genomestudio. Positive values indicate better performance

| DNA input | Phred Filter | AA F1 Without concordance | AA F1 With concordance |
| --- | --- | --- | --- |
| 1 ng | 0 | 99.98% (+0.00%) | 99.96% (+0.01%) |
|  | 10 | 99.98% (+0.00%) | 99.96% (+0.02%) |
|  | 20 | 99.98% (+0.00%) | 99.97% (+0.02%) |
|  | 30 | 99.98% (+0.00%) | 99.98% (+0.03%) |
| 0.5 ng | 0 | 99.61% (+1.56%) | 99.43% (+1.01%) |
|  | 10 | 99.70% (+1.59%) | 99.57% (+1.12%) |
|  | 20 | 99.83% (+1.60%) | 99.78% (+1.29%) |
|  | 30 | 99.90% (+1.58%) | 99.88% (+1.35%) |
| 0.1 ng | 0 | 98.82% (+1.64%) | 98.66% (+1.05%) |
|  | 10 | 99.21% (+1.85%) | 99.13% (+1.42%) |
|  | 20 | 99.65% (+1.94%) | 99.64% (+1.81%) |
|  | 30 | 99.81% (+1.74%) | 99.84% (+1.97%) |
| 0.05 ng | 0 | 98.18% (+1.90%) | 98.09% (+0.81%) |
|  | 10 | 98.47% (+2.07%) | 98.55% (+1.17%) |
|  | 20 | 98.92% (+0.88%) | 99.18% (+1.89%) |
|  | 30 | 99.22% (+2.44%) | 99.45% (+1.76%) |
| 0.01 ng | 0 | 92.80% (+2.00%) | 92.42% (+1.53%) |
|  | 10 | 94.55% (+3.00%) | 95.11% (+3.52%) |
|  | 20 | 97.04% (+4.52%) | 98.12% (+6.70%) |
|  | 30 | 99.05% (+6.96%) | 99.30% (+8.75%) |

Supplemental Table 17. NA7013 F1 Score for the AA class of XGBoost based on 5 individuals in the training set at different DNA input and Phred quality scores.. The percentage point difference in the parenthesis measures the difference between classifier and Genomestudio. Positive values indicate better performance

| DNA input | Phred Filter | BB F1 Without concordance | BB F1 With concordance |
| --- | --- | --- | --- |
| 1 ng | 0 | 99.96% (+0.01%) | 99.96% (+0.01%) |
|  | 10 | 99.96% (+0.02%) | 99.96% (+0.02%) |
|  | 20 | 99.97% (+0.02%) | 99.97% (+0.02%) |
|  | 30 | 99.98% (+0.03%) | 99.98% (+0.03%) |
| 0.5 ng | 0 | 99.43% (+1.01%) | 99.56% (+1.15%) |
|  | 10 | 99.57% (+1.12%) | 99.65% (+1.21%) |
|  | 20 | 99.78% (+1.29%) | 99.81% (+1.31%) |
|  | 30 | 99.88% (+1.35%) | 99.89% (+1.35%) |
| 0.1 ng | 0 | 98.66% (+1.05%) | 99.13% (+1.53%) |
|  | 10 | 99.13% (+1.42%) | 99.35% (+1.64%) |
|  | 20 | 99.64% (+1.81%) | 99.69% (+1.85%) |
|  | 30 | 99.84% (+1.97%) | 99.85% (+1.98%) |
| 0.05 ng | 0 | 98.09% (+0.81%) | 98.38% (+1.09%) |
|  | 10 | 98.55% (+1.17%) | 98.72% (+1.33%) |
|  | 20 | 99.18% (+1.89%) | 99.23% (+1.94%) |
|  | 30 | 99.45% (+1.76%) | 99.47% (+1.77%) |
| 0.01 ng | 0 | 92.42% (+1.53%) | 93.75% (+2.86%) |
|  | 10 | 95.11% (+3.52%) | 95.57% (+3.97%) |
|  | 20 | 98.12% (+6.70%) | 98.20% (+6.78%) |
|  | 30 | 99.30% (+8.75%) | 99.31% (+8.77%) |

Supplemental 18. NA7013 F1 Score for the AB class of XGBoost based based on 5 individuals in the training set at different DNA input and Phred quality scores.The percentage point difference in the parenthesis measures the difference between classifier and Genomestudio. Positive values indicate better performance

| DNA input | Phred Filter | AB F1 Without concordance | AB F1 With concordance |
| --- | --- | --- | --- |
| 1 ng | 0 | 99.84% (+0.04%) | 99.85% (+0.05%) |
|  | 10 | 99.86% (+0.06%) | 99.86% (+0.06%) |
|  | 20 | 99.89% (+0.09%) | 99.89% (+0.09%) |
|  | 30 | 99.91% (+0.10%) | 99.91% (+0.10%) |
| 0.5 ng | 0 | 97.06% (+8.28%) | 97.73% (+8.95%) |
|  | 10 | 97.73% (+8.74%) | 98.18% (+9.19%) |
|  | 20 | 98.81% (+9.42%) | 98.95% (+9.56%) |
|  | 30 | 99.33% (+9.59%) | 99.38% (+9.63%) |
| 0.1 ng | 0 | 91.85% (+10.35%) | 94.30% (+12.80%) |
|  | 10 | 94.41% (+12.40%) | 95.62% (+13.60%) |
|  | 20 | 97.55% (+14.31%) | 97.80% (+14.55%) |
|  | 30 | 98.79% (+14.24%) | 98.86% (+14.32%) |
| 0.05 ng | 0 | 87.00% (+11.78%) | 86.62% (+11.39%) |
|  | 10 | 89.17% (+13.61%) | 88.61% (+13.04%) |
|  | 20 | 92.57% (+16.21%) | 91.93% (+15.57%) |
|  | 30 | 94.45% (+17.25%) | 93.94% (+16.74%) |
| 0.01 ng | 0 | 53.03% (+14.58%) | 47.84% (+9.40%) |
|  | 10 | 58.41% (+19.92%) | 52.36% (+13.87%) |
|  | 20 | 73.33% (+34.53%) | 68.76% (+29.96%) |
|  | 30 | 89.39% (+49.87%) | 87.76% (+48.24%) |

Supplemental Table 19. NA7013 accuracy for XGBoost based on based on 5 individuals in the training set at different DNA input and Phred quality scores. Accuracy is reported with and without a concordance at different DNA inputs and phred quality score filter. The percentage point difference in the parenthesis measures the difference between classifier and Genomestudio. Positive values indicate better performance

| DNA input | Phred Filter | Accuracy Without concordance | Accuracy With concordance |
| --- | --- | --- | --- |
| 1 ng | 0 | 99.95% (+0.01%) | 99.95% (+0.01%) |
|  | 10 | 99.95% (+0.02%) | 99.96% (+0.02%) |
|  | 20 | 99.96% (+0.02%) | 99.96% (+0.02%) |
|  | 30 | 99.97% (+0.03%) | 99.97% (+0.03%) |
| 0.5 ng | 0 | 99.17% (+2.17%) | 99.42% (+2.42%) |
|  | 10 | 99.37% (+2.29%) | 99.54% (+2.46%) |
|  | 20 | 99.67% (+2.47%) | 99.74% (+2.53%) |
|  | 30 | 99.82% (+2.51%) | 99.84% (+2.54%) |
| 0.1 ng | 0 | 97.83% (+2.33%) | 98.73% (+3.23%) |
|  | 10 | 98.56% (+2.84%) | 99.04% (+3.32%) |
|  | 20 | 99.39% (+3.29%) | 99.52% (+3.43%) |
|  | 30 | 99.70% (+3.29%) | 99.75% (+3.34%) |
| 0.05 ng | 0 | 96.78% (+2.34%) | 97.27% (+2.82%) |
|  | 10 | 97.43% (+2.81%) | 97.74% (+3.12%) |
|  | 20 | 98.36% (+3.45%) | 98.48% (+3.57%) |
|  | 30 | 98.84% (+3.68%) | 98.90% (+3.73%) |
| 0.01 ng | 0 | 87.86% (+3.08%) | 89.41% (+4.63%) |
|  | 10 | 91.21% (+5.53%) | 91.77% (+6.09%) |
|  | 20 | 95.72% (+9.64%) | 95.85% (+9.77%) |
|  | 30 | 98.50% (+13.41%) | 98.56% (+13.47%) |

Supplemental Table 20. NA7035 F1 Score for the AA class of XGBoost based on 5 individuals in the training set at different DNA input and Phred quality scores. The percentage point difference in the parenthesis measures the difference between classifier and Genomestudio. Positive values indicate better performance

| DNA input | Phred Filter | AA F1 Without concordance | AA F1 With concordance |
| --- | --- | --- | --- |
| 1 ng | 0 | 99.98% (-0.00%) | 99.94% (+0.02%) |
|  | 10 | 99.98% (+0.00%) | 99.95% (+0.03%) |
|  | 20 | 99.98% (+0.00%) | 99.96% (+0.04%) |
|  | 30 | 99.98% (+0.00%) | 99.97% (+0.04%) |
| 0.5 ng | 0 | 99.50% (+1.40%) | 99.27% (+1.18%) |
|  | 10 | 99.65% (+1.46%) | 99.49% (+1.35%) |
|  | 20 | 99.82% (+1.51%) | 99.78% (+1.58%) |
|  | 30 | 99.90% (+1.50%) | 99.89% (+1.65%) |
| 0.1 ng | 0 | 96.23% (+2.01%) | 92.25% (-0.31%) |
|  | 10 | 97.41% (+2.74%) | 93.79% (+0.94%) |
|  | 20 | 98.99% (+3.52%) | 96.17% (+3.81%) |
|  | 30 | 99.65% (+4.09%) | 97.75% (+6.53%) |
| 0.05 ng | 0 | 97.89% (+1.76%) | 97.38% (+0.57%) |
|  | 10 | 98.19% (+1.93%) | 97.93% (+0.99%) |
|  | 20 | 98.67% (+2.21%) | 98.76% (+1.62%) |
|  | 30 | 98.97% (+2.31%) | 99.19% (+1.93%) |
| 0.01 ng | 0 | 85.67% (+3.52%) | 87.27% (+4.81%) |
|  | 10 | 90.55% (+6.00%) | 92.36% (+7.73%) |
|  | 20 | 97.28% (+11.16%) | 98.01% (+14.17%) |
|  | 30 | 99.28% (+15.49%) | 99.33% (+16.88%) |

Supplemental Table 21. NA7035 F1 Score for the BB class of XGBoost based on 5 individuals in the training set at different DNA input and Phred quality scores. The percentage point difference in the parenthesis measures the difference between classifier and Genomestudio. Positive values indicate better performance

| DNA input | Phred Filter | BB F1 Without concordance | BB F1 With concordance |
| --- | --- | --- | --- |
| 1 ng | 0 | 99.94% (+0.02%) | 99.95% (+0.03%) |
|  | 10 | 99.95% (+0.03%) | 99.96% (+0.03%) |
|  | 20 | 99.96% (+0.04%) | 99.96% (+0.04%) |
|  | 30 | 99.97% (+0.04%) | 99.97% (+0.04%) |
| 0.5 ng | 0 | 99.27% (+1.18%) | 99.51% (+1.42%) |
|  | 10 | 99.49% (+1.35%) | 99.64% (+1.50%) |
|  | 20 | 99.78% (+1.58%) | 99.81% (+1.61%) |
|  | 30 | 99.89% (+1.65%) | 99.89% (+1.65%) |
| 0.1 ng | 0 | 92.25% (-0.31%) | 93.94% (+1.38%) |
|  | 10 | 93.79% (+0.94%) | 94.72% (+1.87%) |
|  | 20 | 96.17% (+3.81%) | 96.44% (+4.07%) |
|  | 30 | 97.75% (+6.53%) | 97.80% (+6.58%) |
| 0.05 ng | 0 | 97.38% (+0.57%) | 97.92% (+1.11%) |
|  | 10 | 97.93% (+0.99%) | 98.25% (+1.32%) |
|  | 20 | 98.76% (+1.62%) | 98.85% (+1.71%) |
|  | 30 | 99.19% (+1.93%) | 99.22% (+1.95%) |
| 0.01 ng | 0 | 87.27% (+4.81%) | 89.03% (+6.57%) |
|  | 10 | 92.36% (+7.73%) | 92.89% (+8.26%) |
|  | 20 | 98.01% (+14.17%) | 98.19% (+14.36%) |
|  | 30 | 99.33% (+16.88%) | 99.36% (+16.90%) |

Supplemental 22. NA7035 F1 Score for the AB class of XGBoost based on 5 individuals in the training set at different DNA input and Phred quality scores. The percentage point difference in the parenthesis measures the difference between classifier and Genomestudio. Positive values indicate better performance

| DNA input | Phred Filter | AB F1 Without concordance | AB F1 With concordance |
| --- | --- | --- | --- |
| 1 ng | 0 | 99.77% (+0.07%) | 99.81% (+0.10%) |
|  | 10 | 99.80% (+0.10%) | 99.83% (+0.12%) |
|  | 20 | 99.86% (+0.14%) | 99.86% (+0.15%) |
|  | 30 | 99.88% (+0.16%) | 99.88% (+0.16%) |
| 0.5 ng | 0 | 96.26% (+8.45%) | 97.44% (+9.64%) |
|  | 10 | 97.30% (+9.20%) | 98.07% (+9.97%) |
|  | 20 | 98.71% (+10.18%) | 98.93% (+10.40%) |
|  | 30 | 99.32% (+10.40%) | 99.38% (+10.46%) |
| 0.1 ng | 0 | 68.13% (+9.04%) | 69.11% (+10.02%) |
|  | 10 | 73.30% (+13.98%) | 72.63% (+13.31%) |
|  | 20 | 83.66% (+23.94%) | 82.12% (+22.40%) |
|  | 30 | 90.77% (+30.53%) | 89.67% (+29.43%) |
| 0.05 ng | 0 | 83.18% (+10.74%) | 83.18% (+10.74%) |
|  | 10 | 85.53% (+12.77%) | 85.07% (+12.31%) |
|  | 20 | 89.66% (+16.13%) | 88.78% (+15.24%) |
|  | 30 | 92.21% (+17.86%) | 91.43% (+17.08%) |
| 0.01 ng | 0 | 31.73% (+9.72%) | 27.35% (+5.35%) |
|  | 10 | 29.28% (+8.68%) | 24.26% (+3.67%) |
|  | 20 | 50.19% (+32.00%) | 44.95% (+26.75%) |
|  | 30 | 77.53% (+60.09%) | 74.89% (+57.45%) |

Supplemental Table 23. NA7035 accuracy for XGBoost based on based on 5 individuals in the training set at different DNA input and Phred quality scores. Accuracy is reported with and without a concordance at different DNA inputs and phred quality score filters. The percentage point difference in the parenthesis measures the difference between classifier and Genomestudio. Positive values indicate better performance

| DNA input | Phred Filter | Accuracy Without concordance | Accuracy With concordance |
| --- | --- | --- | --- |
| 1 ng | 0 | 99.93% (+0.02%) | 99.94% (+0.03%) |
|  | 10 | 99.94% (+0.03%) | 99.95% (+0.03%) |
|  | 20 | 99.96% (+0.04%) | 99.96% (+0.04%) |
|  | 30 | 99.96% (+0.05%) | 99.96% (+0.05%) |
| 0.5 ng | 0 | 98.95% (+2.23%) | 99.35% (+2.63%) |
|  | 10 | 99.26% (+2.42%) | 99.52% (+2.69%) |
|  | 20 | 99.65% (+2.69%) | 99.74% (+2.77%) |
|  | 30 | 99.82% (+2.74%) | 99.85% (+2.77%) |
| 0.1 ng | 0 | 90.00% (+1.22%) | 92.35% (+3.57%) |
|  | 10 | 92.19% (+2.95%) | 93.47% (+4.23%) |
|  | 20 | 95.58% (+6.14%) | 95.89% (+6.45%) |
|  | 30 | 97.59% (+8.70%) | 97.64% (+8.76%) |
| 0.05 ng | 0 | 95.86% (+2.00%) | 96.58% (+2.72%) |
|  | 10 | 96.58% (+2.53%) | 97.04% (+2.98%) |
|  | 20 | 97.74% (+3.35%) | 97.88% (+3.50%) |
|  | 30 | 98.39% (+3.75%) | 98.44% (+3.79%) |
| 0.01 ng | 0 | 80.36% (+6.50%) | 82.17% (+8.30%) |
|  | 10 | 86.53% (+10.30%) | 87.00% (+10.77%) |
|  | 20 | 96.00% (+20.05%) | 96.22% (+20.26%) |
|  | 30 | 98.77% (+25.71%) | 98.82% (+25.76%) |

Supplemental Table 24. Kinship coefficient between 7046 and the other individuals from the pedigree. The genotypes from GenomeStudio and the model were used to generate the kinship coefficient. There appears to be an improvement over GenomeStudio.

| SamplePair1 | SamplePair2 | KinshipCoefficient |
| --- | --- | --- |
| 13047_50_Ng | 7046_0.01_Ng | -0.72891 |
| 13047_50_Ng | 7046_0.01_Ng_Ml_threshold_0 | -0.76777 |
| 13047_50_Ng | 7046_0.01_Ng_Ml_threshold_10 | -0.54904 |
| 13047_50_Ng | 7046_0.01_Ng_Ml_threshold_20 | -1.0749 |
| 13047_50_Ng | 7046_0.01_Ng_Ml_threshold_30 | -1.62927 |
| 13047_50_Ng | 7046_0.05_Ng | 0.080028 |
| 13047_50_Ng | 7046_0.05_Ng_Ml_threshold_0 | 0.123003 |
| 13047_50_Ng | 7046_0.05_Ng_Ml_threshold_10 | 0.138082 |
| 13047_50_Ng | 7046_0.05_Ng_Ml_threshold_20 | 0.167343 |
| 13047_50_Ng | 7046_0.05_Ng_Ml_threshold_30 | 0.164189 |
| 13047_50_Ng | 7046_0.1_Ng | 0.126961 |
| 13047_50_Ng | 7046_0.1_Ng_Ml_threshold_0 | 0.148734 |
| 13047_50_Ng | 7046_0.1_Ng_Ml_threshold_10 | 0.164865 |
| 13047_50_Ng | 7046_0.1_Ng_Ml_threshold_20 | 0.196992 |
| 13047_50_Ng | 7046_0.1_Ng_Ml_threshold_30 | 0.213592 |
| 13047_50_Ng | 7046_0.5_Ng | 0.148106 |
| 13047_50_Ng | 7046_0.5_Ng_Ml_threshold_0 | 0.181667 |
| 13047_50_Ng | 7046_0.5_Ng_Ml_threshold_10 | 0.199951 |
| 13047_50_Ng | 7046_0.5_Ng_Ml_threshold_20 | 0.223301 |
| 13047_50_Ng | 7046_0.5_Ng_Ml_threshold_30 | 0.231733 |
| 13047_50_Ng | 7046_1.0_Ng | 0.242124 |
| 13047_50_Ng | 7046_1.0_Ng_Ml_threshold_0 | 0.242755 |
| 13047_50_Ng | 7046_1.0_Ng_Ml_threshold_10 | 0.242876 |
| 13047_50_Ng | 7046_1.0_Ng_Ml_threshold_20 | 0.243172 |
| 13047_50_Ng | 7046_1.0_Ng_Ml_threshold_30 | 0.243405 |
| 13047_50_Ng | 7046_50_Ng | 0.24297 |
| 13050_50_Ng | 7046_0.01_Ng | -0.67916 |
| 13050_50_Ng | 7046_0.01_Ng_Ml_threshold_0 | -0.71613 |
| 13050_50_Ng | 7046_0.01_Ng_Ml_threshold_10 | -0.54476 |
| 13050_50_Ng | 7046_0.01_Ng_Ml_threshold_20 | -1.06813 |
| 13050_50_Ng | 7046_0.01_Ng_Ml_threshold_30 | -1.61827 |
| 13050_50_Ng | 7046_0.05_Ng | 0.082564 |
| 13050_50_Ng | 7046_0.05_Ng_Ml_threshold_0 | 0.135339 |
| 13050_50_Ng | 7046_0.05_Ng_Ml_threshold_10 | 0.149868 |
| 13050_50_Ng | 7046_0.05_Ng_Ml_threshold_20 | 0.169377 |
| 13050_50_Ng | 7046_0.05_Ng_Ml_threshold_30 | 0.164933 |
| 13050_50_Ng | 7046_0.1_Ng | 0.13544 |
| 13050_50_Ng | 7046_0.1_Ng_Ml_threshold_0 | 0.159431 |
| 13050_50_Ng | 7046_0.1_Ng_Ml_threshold_10 | 0.174833 |
| 13050_50_Ng | 7046_0.1_Ng_Ml_threshold_20 | 0.205978 |
| 13050_50_Ng | 7046_0.1_Ng_Ml_threshold_30 | 0.214359 |
| 13050_50_Ng | 7046_0.5_Ng | 0.160076 |
| 13050_50_Ng | 7046_0.5_Ng_Ml_threshold_0 | 0.190121 |
| 13050_50_Ng | 7046_0.5_Ng_Ml_threshold_10 | 0.208091 |
| 13050_50_Ng | 7046_0.5_Ng_Ml_threshold_20 | 0.230956 |
| 13050_50_Ng | 7046_0.5_Ng_Ml_threshold_30 | 0.239861 |
| 13050_50_Ng | 7046_1.0_Ng | 0.245737 |
| 13050_50_Ng | 7046_1.0_Ng_Ml_threshold_0 | 0.245322 |
| 13050_50_Ng | 7046_1.0_Ng_Ml_threshold_10 | 0.245364 |
| 13050_50_Ng | 7046_1.0_Ng_Ml_threshold_20 | 0.245337 |
| 13050_50_Ng | 7046_1.0_Ng_Ml_threshold_30 | 0.245111 |
| 13050_50_Ng | 7046_50_Ng | 0.248738 |
| 13053_50_Ng | 7046_0.01_Ng | -0.82612 |
| 13053_50_Ng | 7046_0.01_Ng_Ml_threshold_0 | -0.85686 |
| 13053_50_Ng | 7046_0.01_Ng_Ml_threshold_10 | -0.78265 |
| 13053_50_Ng | 7046_0.01_Ng_Ml_threshold_20 | -1.60348 |
| 13053_50_Ng | 7046_0.01_Ng_Ml_threshold_30 | -2.45273 |
| 13053_50_Ng | 7046_0.05_Ng | -0.18761 |
| 13053_50_Ng | 7046_0.05_Ng_Ml_threshold_0 | -0.0934 |
| 13053_50_Ng | 7046_0.05_Ng_Ml_threshold_10 | -0.08006 |
| 13053_50_Ng | 7046_0.05_Ng_Ml_threshold_20 | -0.09343 |
| 13053_50_Ng | 7046_0.05_Ng_Ml_threshold_30 | -0.12339 |
| 13053_50_Ng | 7046_0.1_Ng | -0.12932 |
| 13053_50_Ng | 7046_0.1_Ng_Ml_threshold_0 | -0.07205 |
| 13053_50_Ng | 7046_0.1_Ng_Ml_threshold_10 | -0.05796 |
| 13053_50_Ng | 7046_0.1_Ng_Ml_threshold_20 | -0.02989 |
| 13053_50_Ng | 7046_0.1_Ng_Ml_threshold_30 | -0.05024 |
| 13053_50_Ng | 7046_0.5_Ng | -0.08599 |
| 13053_50_Ng | 7046_0.5_Ng_Ml_threshold_0 | -0.04271 |
| 13053_50_Ng | 7046_0.5_Ng_Ml_threshold_10 | -0.02648 |
| 13053_50_Ng | 7046_0.5_Ng_Ml_threshold_20 | -0.00379 |
| 13053_50_Ng | 7046_0.5_Ng_Ml_threshold_30 | 0.002959 |
| 13053_50_Ng | 7046_1.0_Ng | -0.00787 |
| 13053_50_Ng | 7046_1.0_Ng_Ml_threshold_0 | -0.00969 |
| 13053_50_Ng | 7046_1.0_Ng_Ml_threshold_10 | -0.00978 |
| 13053_50_Ng | 7046_1.0_Ng_Ml_threshold_20 | -0.01023 |
| 13053_50_Ng | 7046_1.0_Ng_Ml_threshold_30 | -0.01102 |
| 13053_50_Ng | 7046_50_Ng | -0.00167 |
| 7013_50_Ng | 7046_0.01_Ng | -0.85116 |
| 7013_50_Ng | 7046_0.01_Ng_Ml_threshold_0 | -0.88091 |
| 7013_50_Ng | 7046_0.01_Ng_Ml_threshold_10 | -0.78113 |
| 7013_50_Ng | 7046_0.01_Ng_Ml_threshold_20 | -1.597 |
| 7013_50_Ng | 7046_0.01_Ng_Ml_threshold_30 | -2.44208 |
| 7013_50_Ng | 7046_0.05_Ng | -0.18603 |
| 7013_50_Ng | 7046_0.05_Ng_Ml_threshold_0 | -0.09901 |
| 7013_50_Ng | 7046_0.05_Ng_Ml_threshold_10 | -0.08569 |
| 7013_50_Ng | 7046_0.05_Ng_Ml_threshold_20 | -0.0917 |
| 7013_50_Ng | 7046_0.05_Ng_Ml_threshold_30 | -0.12116 |
| 7013_50_Ng | 7046_0.1_Ng | -0.1248 |
| 7013_50_Ng | 7046_0.1_Ng_Ml_threshold_0 | -0.07704 |
| 7013_50_Ng | 7046_0.1_Ng_Ml_threshold_10 | -0.06246 |
| 7013_50_Ng | 7046_0.1_Ng_Ml_threshold_20 | -0.03368 |
| 7013_50_Ng | 7046_0.1_Ng_Ml_threshold_30 | -0.04756 |
| 7013_50_Ng | 7046_0.5_Ng | -0.09017 |
| 7013_50_Ng | 7046_0.5_Ng_Ml_threshold_0 | -0.04821 |
| 7013_50_Ng | 7046_0.5_Ng_Ml_threshold_10 | -0.03186 |
| 7013_50_Ng | 7046_0.5_Ng_Ml_threshold_20 | -0.00855 |
| 7013_50_Ng | 7046_0.5_Ng_Ml_threshold_30 | 0.005199 |
| 7013_50_Ng | 7046_1.0_Ng | -0.00515 |
| 7013_50_Ng | 7046_1.0_Ng_Ml_threshold_0 | -0.00702 |
| 7013_50_Ng | 7046_1.0_Ng_Ml_threshold_10 | -0.00711 |
| 7013_50_Ng | 7046_1.0_Ng_Ml_threshold_20 | -0.00761 |
| 7013_50_Ng | 7046_1.0_Ng_Ml_threshold_30 | -0.00847 |
| 7013_50_Ng | 7046_50_Ng | 0.001073 |
| 7028_50_Ng | 7046_0.01_Ng | -0.67877 |
| 7028_50_Ng | 7046_0.01_Ng_Ml_threshold_0 | -0.71731 |
| 7028_50_Ng | 7046_0.01_Ng_Ml_threshold_10 | -0.54343 |
| 7028_50_Ng | 7046_0.01_Ng_Ml_threshold_20 | -1.07028 |
| 7028_50_Ng | 7046_0.01_Ng_Ml_threshold_30 | -1.62876 |
| 7028_50_Ng | 7046_0.05_Ng | 0.081348 |
| 7028_50_Ng | 7046_0.05_Ng_Ml_threshold_0 | 0.131448 |
| 7028_50_Ng | 7046_0.05_Ng_Ml_threshold_10 | 0.146423 |
| 7028_50_Ng | 7046_0.05_Ng_Ml_threshold_20 | 0.169526 |
| 7028_50_Ng | 7046_0.05_Ng_Ml_threshold_30 | 0.165174 |
| 7028_50_Ng | 7046_0.1_Ng | 0.131401 |
| 7028_50_Ng | 7046_0.1_Ng_Ml_threshold_0 | 0.155259 |
| 7028_50_Ng | 7046_0.1_Ng_Ml_threshold_10 | 0.171037 |
| 7028_50_Ng | 7046_0.1_Ng_Ml_threshold_20 | 0.20299 |
| 7028_50_Ng | 7046_0.1_Ng_Ml_threshold_30 | 0.213425 |
| 7028_50_Ng | 7046_0.5_Ng | 0.154883 |
| 7028_50_Ng | 7046_0.5_Ng_Ml_threshold_0 | 0.18809 |
| 7028_50_Ng | 7046_0.5_Ng_Ml_threshold_10 | 0.206191 |
| 7028_50_Ng | 7046_0.5_Ng_Ml_threshold_20 | 0.229354 |
| 7028_50_Ng | 7046_0.5_Ng_Ml_threshold_30 | 0.238438 |
| 7028_50_Ng | 7046_1.0_Ng | 0.244856 |
| 7028_50_Ng | 7046_1.0_Ng_Ml_threshold_0 | 0.244454 |
| 7028_50_Ng | 7046_1.0_Ng_Ml_threshold_10 | 0.244489 |
| 7028_50_Ng | 7046_1.0_Ng_Ml_threshold_20 | 0.244455 |
| 7028_50_Ng | 7046_1.0_Ng_Ml_threshold_30 | 0.244226 |
| 7028_50_Ng | 7046_50_Ng | 0.24781 |
| 7035_50_Ng | 7046_0.01_Ng | -0.775 |
| 7035_50_Ng | 7046_0.01_Ng_Ml_threshold_0 | -0.80462 |
| 7035_50_Ng | 7046_0.01_Ng_Ml_threshold_10 | -0.66062 |
| 7035_50_Ng | 7046_0.01_Ng_Ml_threshold_20 | -1.32658 |
| 7035_50_Ng | 7046_0.01_Ng_Ml_threshold_30 | -2.01955 |
| 7035_50_Ng | 7046_0.05_Ng | -0.05119 |
| 7035_50_Ng | 7046_0.05_Ng_Ml_threshold_0 | 0.01539 |
| 7035_50_Ng | 7046_0.05_Ng_Ml_threshold_10 | 0.029291 |
| 7035_50_Ng | 7046_0.05_Ng_Ml_threshold_20 | 0.04269 |
| 7035_50_Ng | 7046_0.05_Ng_Ml_threshold_30 | 0.026777 |
| 7035_50_Ng | 7046_0.1_Ng | 0.005806 |
| 7035_50_Ng | 7046_0.1_Ng_Ml_threshold_0 | 0.039348 |
| 7035_50_Ng | 7046_0.1_Ng_Ml_threshold_10 | 0.054345 |
| 7035_50_Ng | 7046_0.1_Ng_Ml_threshold_20 | 0.084413 |
| 7035_50_Ng | 7046_0.1_Ng_Ml_threshold_30 | 0.087425 |
| 7035_50_Ng | 7046_0.5_Ng | 0.033692 |
| 7035_50_Ng | 7046_0.5_Ng_Ml_threshold_0 | 0.070764 |
| 7035_50_Ng | 7046_0.5_Ng_Ml_threshold_10 | 0.087908 |
| 7035_50_Ng | 7046_0.5_Ng_Ml_threshold_20 | 0.111076 |
| 7035_50_Ng | 7046_0.5_Ng_Ml_threshold_30 | 0.122549 |
| 7035_50_Ng | 7046_1.0_Ng | 0.123208 |
| 7035_50_Ng | 7046_1.0_Ng_Ml_threshold_0 | 0.122175 |
| 7035_50_Ng | 7046_1.0_Ng_Ml_threshold_10 | 0.122161 |
| 7035_50_Ng | 7046_1.0_Ng_Ml_threshold_20 | 0.121926 |
| 7035_50_Ng | 7046_1.0_Ng_Ml_threshold_30 | 0.121433 |
| 7035_50_Ng | 7046_50_Ng | 0.125614 |
| 7437_50_Ng | 7046_0.01_Ng | -0.66772 |
| 7437_50_Ng | 7046_0.01_Ng_Ml_threshold_0 | -0.7009 |
| 7437_50_Ng | 7046_0.01_Ng_Ml_threshold_10 | -0.5461 |
| 7437_50_Ng | 7046_0.01_Ng_Ml_threshold_20 | -1.07835 |
| 7437_50_Ng | 7046_0.01_Ng_Ml_threshold_30 | -1.63123 |
| 7437_50_Ng | 7046_0.05_Ng | 0.078202 |
| 7437_50_Ng | 7046_0.05_Ng_Ml_threshold_0 | 0.133162 |
| 7437_50_Ng | 7046_0.05_Ng_Ml_threshold_10 | 0.147242 |
| 7437_50_Ng | 7046_0.05_Ng_Ml_threshold_20 | 0.167988 |
| 7437_50_Ng | 7046_0.05_Ng_Ml_threshold_30 | 0.164217 |
| 7437_50_Ng | 7046_0.1_Ng | 0.131413 |
| 7437_50_Ng | 7046_0.1_Ng_Ml_threshold_0 | 0.159416 |
| 7437_50_Ng | 7046_0.1_Ng_Ml_threshold_10 | 0.174432 |
| 7437_50_Ng | 7046_0.1_Ng_Ml_threshold_20 | 0.205005 |
| 7437_50_Ng | 7046_0.1_Ng_Ml_threshold_30 | 0.21389 |
| 7437_50_Ng | 7046_0.5_Ng | 0.160379 |
| 7437_50_Ng | 7046_0.5_Ng_Ml_threshold_0 | 0.19192 |
| 7437_50_Ng | 7046_0.5_Ng_Ml_threshold_10 | 0.209059 |
| 7437_50_Ng | 7046_0.5_Ng_Ml_threshold_20 | 0.231184 |
| 7437_50_Ng | 7046_0.5_Ng_Ml_threshold_30 | 0.239835 |
| 7437_50_Ng | 7046_1.0_Ng | 0.245049 |
| 7437_50_Ng | 7046_1.0_Ng_Ml_threshold_0 | 0.244635 |
| 7437_50_Ng | 7046_1.0_Ng_Ml_threshold_10 | 0.244688 |
| 7437_50_Ng | 7046_1.0_Ng_Ml_threshold_20 | 0.244652 |
| 7437_50_Ng | 7046_1.0_Ng_Ml_threshold_30 | 0.24446 |
| 7437_50_Ng | 7046_50_Ng | 0.247587 |
